# Optic nerve as a source of activated retinal microglia post-injury

**DOI:** 10.1186/s40478-018-0571-8

**Published:** 2018-07-23

**Authors:** Neal D. Heuss, Mark J. Pierson, Heidi Roehrich, Scott W. McPherson, Andrea L. Gram, Ling Li, Dale S. Gregerson

**Affiliations:** 1 Department of Ophthalmology and Visual Neurosciences, University of Minnesota, Minneapolis, MN USA; 2 Department of Experimental and Clinical Pharmacology, University of Minnesota, Minneapolis, MN USA

**Keywords:** Retina, Optic nerve, Microglia, Injury response, Migration, Origin

## Abstract

Using mice expressing green fluorescent protein (GFP) from a transgenic CD11c promoter we found that a controlled optic nerve crush (ONC) injury attracted GFP^hi^ retinal myeloid cells to the dying retinal ganglion cells and their axons. However, the origin of these retinal myeloid cells was uncertain. In this study we use transgenic mice in conjunction with ONC, partial and full optic nerve transection (ONT), and parabiosis to determine the origin of injury induced retinal myeloid cells. Analysis of parabiotic mice and fate mapping showed that responding retinal myeloid cells were not derived from circulating macrophages and that GFP^hi^ myeloid cells could be derived from GFP^lo^ microglia. Comparison of optic nerve to retina following an ONC showed a much greater concentration of GFP^hi^ cells and GFP^lo^ microglia in the optic nerve. Optic nerve injury also induced Ki67^+^ cells in the optic nerve but not in the retina. Comparison of the retinal myeloid cell response after full versus partial ONT revealed fewer GFP^hi^ cells and GFP^lo^ microglia in the retina following a full ONT despite it being a more severe injury, suggesting that full transection of the optic nerve can block the migration of responding myeloid cells to the retina. Our results suggest that the optic nerve can be a reservoir for activated microglia and other retinal myeloid cells in the retina following optic nerve injury.

**Electronic supplementary material:**

The online version of this article (10.1186/s40478-018-0571-8) contains supplementary material, which is available to authorized users.

## Introduction

Microglia are the major population of immune cells in quiescent retina and are important for maintenance of retinal homeostasis [53]. The microglia niche is filled by the initial seeding of CNS with yolk sac progenitors during development [14]. Although microglia are widely reported to be the antigen presenting cells (APC) of the CNS, very few reports included definitive functional assays; i.e. antigen processing that supported generation of cognate peptides for presentation to naïve antigen-specific T cells [4, 47, 58]. Our experiments testing murine retinal microglia found that they did not fulfill these critical functional requirements for antigen presentation [17]. Since dendritic cells are the classic “professional” APC and express CD11c, we tested the CD11c^GFP^ reporter mice [27] in our search for retinal APC. GFP reporter expression in retina revealed a population of microglia-like cells (CD45^med^CD11b^hi^Iba1^+^F4/80^+^Ly6C^lo^CX3CR1^hi^) that expressed GFP (GFP^hi^ cells) from the transgenic CD11c promoter [33, 62]. We found by in vitro and in vivo studies that GFP expression in these retinal cells correlated with APC function [17, 18, 46].

Our studies of the APC function of these GFP^hi^ retinal myeloid cells in CD11c^GFP^ reporter mice showed that an optic nerve crush (ONC) injury to the axons of retinal ganglion cells (RGC) generated large numbers of retinal GFP^hi^ myeloid cells [33]. These cells were then found to dominate the clearance of RGC/axon debris after an ONC [19]. Recently we reported that the GFP^hi^ myeloid cells in CD11c^GFP^ mouse retina were major responders in the outer retina during cone photoreceptor degeneration in RPE65 knockout mice. The evidence suggested that they were an activated form of microglia [62], and no evidence of recruited myeloid cells was found in this degeneration model.

Studies using radiation-bone marrow (BM) chimeric mice given a retinal injury after BM grafting showed that rapid recruitment of circulating BM-derived macrophages comprised the injury response [29]. In the absence of injury, the retina was slowly repopulated with microglia-like macrophages derived from the donor BM [67]. However, studies in brain using parabiosis in place of radiation-BM chimeras showed that circulating precursors of CNS myeloid cells were not recruited by injuries in the absence of the radiation chimerism protocol [1, 31]. Prior studies concerning the presence and origin of myeloid cells in the optic nerve, before and after injury to the optic nerve or retina, provided evidence that circulating macrophages were readily recruited into the optic nerve and optic nerve head (ONH) [26, 32]. Others suggested that the optic nerve could be a source of retinal myeloid cells [23, 26]. These differing results raised the possibility that microglia could traffic from optic nerve to retina but were complicated by the use of radiation-BM chimeras. The origin of the injury-induced GFP^hi^ myeloid cells in CD11c^GFP^ mouse retina was of interest, as they dominated the response in the retinal ganglion cell (RGC)/nerve fiber layer (NFL) post-ONC [33].

In preliminary studies we found that the microglia in optic nerve responded vigorously to injury and rapidly repopulated following ablation, unlike the retina. To further explore the origin of the injury-induced retinal GFP^hi^ myeloid cells in this reporter mouse strain, we examined the possibility that they originated from microglia in retina and optic nerve. Microglia proliferation has been reported in various CNS stresses and injuries [6, 16, 64], including retina following an optic nerve transection (ONT) [66]. Evidence for production of microglia by local progenitors in brain [9] and retina [65] has been found, as well as migration from other regions contributing to accumulation at the site of injury [40, 57]. While acknowledging the observations that retinal microglial proliferation post-ablation may account for a major part of the retinal repopulation response [23, 24, 71], we found evidence that optic nerve was a source of retinal myeloid cells following optic nerve injury in an already fully-populated retina.

## Materials and methods

### Mice

Nomenclature and sources of the mice are listed in Table 1. CD11c^GFP^ mice express eGFP as a chimeric cell membrane protein comprised of the diphtheria toxin receptor and GFP under control of a transgenic CD11c promoter [27]. It is important to note that the transgenic CD11c promoter driving GFP expression is not the same as the endogenous CD11c promoter, and that expression of the endogenous CD11c protein in retinal myeloid cells does not correlate with expression of the GFP reporter in retina [33]. CD11c^GFP^ mice crossed with CX3CR1^YFP-creER^ mice were also used to examine injury-induced transgenic GFP expression in microglia in combination with expression of other common markers of microglia including CD11b and/or F4/80. CD11c^GFP^ mice were also crossed with the R161H mice that develop spontaneous autoimmune uveoretinitis [20, 21]. The retinal inflammation in CD11c^GFP^:R161H mice provided positive controls for Ki67 staining of proliferating immune cells in inflamed retina. Since CD4 T cell antigen recognition in the R161H T cells is B10.R3-restricted, breeding was done to generate these mice on the (B10.R3 x B6J)_F1_ background. Briefly, R161H mice on the B10.R3 background were mated with CD11c^GFP^ mice (B6J background) to produce F_1_ offspring. F_1_ pups expressing the CD11c^GFP^ transgene and the R161H T cell antigen receptor spontaneously developed autoimmune uveoretinitis. ACTb^eGFP^ mice express GFP in many cells driven by a βactin promoter and were used to track donor cells in recipient mice in parabiosis experiments. CX3CR1^YFP-creER^ mice were also crossed with floxed Tomato Red reporter mice (R26^RFP^) and CD11c^GFP^ mice for fate mapping. Tamoxifen (Tam) was used to activate cre in cells expressing CX3CR1 promoted YFP-creER, inducing RFP expression in those cells. All mice were *rd8*-negative [45]. Mice were reared under cyclic light in specific pathogen-free conditions. Mice were sacrificed by CO_2_ exposure.

**Table 1:** Mice and nomenclature

Common name^a^	Stock number^b^	Strain name^b^	Refs
CX3CR1^YFP-creER^	021160	B6.129P2(Cg)-*Cx3cr1*^*tm2.1(cre/ERT2)Litt*^/WganJ	[51]
CD11c^GFP^	004509	B6.FVB-Tg(Itgax-DTR/EGFP)57Lan/J	[27]
R26^RFP^	007914	B6.Cg-*Gt(ROSA)26Sor*^*tm14(CAG-tdTomato)Hze*^/J	[44]
ACTb^GFP^	003291	C57BL/6-Tg(CAG-EGFP)1Osb/J	[49]
B6J	000664	C57BL/6 J	
R161H	none	R161H mice (B10.R3 background), obtained from Dr. Rachel Caspi, NEI/NIH	[20, 21]

^a^Used in text. ^b^Jackson Labs

### Fate mapping the origin of retinal GFP^hi^ myeloid cells

Fate mapping strategies from the Saban lab and others [15, 30, 50, 51] were adapted to examine the origins of the retinal GFP^hi^ myeloid cells. The CX3CR1^YFP-CreER^:R26^RFP^ mice were crossed with CD11c^GFP^ mice for these experiments. Tam was given twice on alternate days at the 3 mg/dose as previously described [62] so that CX3CR1^+^ cells upregulated expression of RFP. At 70 days post-Tam, mice were given an optic nerve crush. Eight days later the mice were examined for induction of GFP-expression in the RFP^+^ retinal myeloid cells.

### Optic nerve transection (ONT)

To sever the optic nerve and preserve the ophthalmic artery and blood flow to the retina, an ONT was done one mm from the posterior pole. The optic nerve of the left eye was exposed using the same strategy used for the optic nerve crush [35]. Once exposed, the nerve was cut using #15003–08 Vannas-Tubingen Straight Spring Scissors. The procedure was delicate but allowed the critical preservation of blood flow to the retina while cutting all or part of the nerve.

### Optic nerve crush (ONC)

**Fig. 1:**
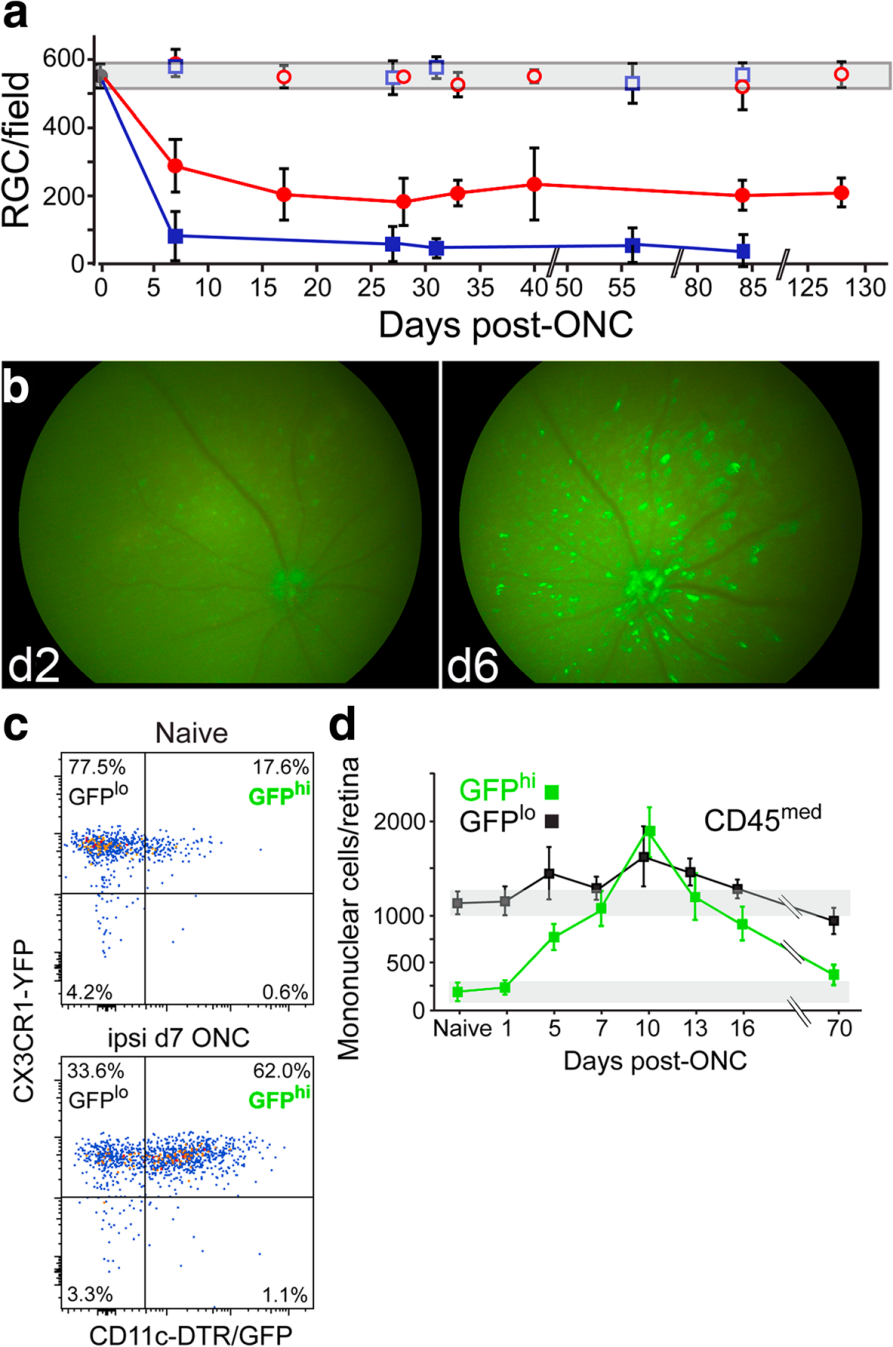
An optic nerve crush (ONC) injury stimulated appearance of GFP^hi^YFP^hi^ myeloid cells in the retina of CX3CR1^YFP-creER^:CD11c^GFP^ mice. **a** Time course of post-ONC loss of RGC comparing self-closing forceps with DSAEK forceps. Gray shaded area at top of graph represents the normal number of RGC/field ±1 SD. Area of field = 0.19 mm^2^. (Red: DSAEK forceps; Blue: #N7 self-closing forceps.) **b** Fluorescence fundus photos detecting the expression of GFP from GFP^hi^ myeloid cells. The same retina at 2 days and 6 days post-ONC is shown. **c** Representative flow cytometry results of analyses of GFP^hi^ and GFP^lo^ populations of retinal myeloid cells (gated on viable, doublet-excluded CD45^med^CD11b^+^Ly6G^−^ cells). **d** Time course of the appearance of GFP^hi^ myeloid cells and GFP^lo^ microglia in retina after an acute ONC injury. Average of 4–9 retinas per time point

An ONC was used in several experiments to reproducibly injure the optic nerve with very little risk to the ophthalmic artery and blood flow. This procedure used #2197E DSAEK forceps, not self-closing (Ambler Surgical), to limit severity of the crush [19, 33]. Approximately 50% of ganglion cells die via apoptosis by 15 days following this ONC procedure, reaching a stable plateau for at least 4 months. Its use was validated relative to the often-used #N7 Dumont self-closing forceps procedure as shown in Fig. 1.

### Flow cytometry

Mice were euthanized, perfused, and the retinas removed as described [19, 33, 46, 62]. Retinas were suspended in 0.5 mg/ml Liberase/TM (Roche) and 0.05% DNase in DPBS and gently dispersed by trituration. Fluorescent-labeled antibodies (BD Biosciences and eBioscience) and viability dye (eFluor 780 Fixable Viability Dye, eBioscience) were added to cell suspensions and incubated on ice for 30 min to assess CD45, CD11b and Ly6G. Samples containing GFP and YFP reporters were excited using a 488 nm laser and detected using 550/30 (YFP) and 510/21 (GFP) bandpass filters separated by a 525 nm longpass mirror [63]. RFP was excited using a 561 nm laser and detected using a 585/15 filter set. Fluorescent proteins were compensated with single color and fluorescence minus one (FMO) controls. Data analysis was done with FlowJo (Tree Star) software. Data collected from a single retina comprised a single sample. Samples of optic nerve, 4–5 mm in length, were similarly prepared and analyzed as a single sample. Gating strategy for flow counting retina, brain, and optic nerve samples was based on selection of all CD45^+^ cells, viable CD45^+^ cells, doublet rejection by FSC-height vs FCS-area scatter analysis, followed by gating on CD45^med^CD11b^hi^Ly6G^−^ for mononuclear cells. Blood samples were stained with the appropriate antibodies, lysed in 0.17 M NH_4_Cl, washed and resuspended in DPBS with 2% fetal bovine serum and then analyzed with monocytes being identified as CD45^+^CD11b^+^Ly6G^−^.

### Retina flat mounts

Retina flatmounts were prepared as previously described [61]. Isolated retina was fixed in 4% paraformaldehyde (PFA) for 5–10 min at room temperature (RT). Retinas were washed with PBS, blocked with 10% normal donkey serum for 1 h, flattened by four radial relaxing cuts, and stained. Retinas were incubated with primary antibodies and washed 6 times in PBS. Secondary antibodies were incubated for 3 h, washed 6 times with PBS, and coverslipped. Samples were mounted with DAPI (Immu-Mount, Vectashield, Burlingame, CA) or Fluoromount-G (Southern Biotech, Birmingham, AL) for fluorescence microscopy.

### Fundus imaging

Mice were anesthetized with a solution of 50 mg/mL ketamine (Akorn, Lake Forest, IL) and 5 mg/ml xylazine (Lloyd Laboratories, Shenandoah, IA) using 2 μl/g mouse. Pupil dilation was done with 2.5 μl of (0.5%) tropicamide (Bausch and Lomb, Tampa FL) and (0.25%) proparacaine (Akorn) solution applied topically to the cornea. Corneal hydration was maintained by liberal application of Systane (Alcon, Fort Worth, TX) or GenTeal (Alcon). Retinal images were obtained using a Micron III retinal imaging microscope (Phoenix Research Laboratories, Pleasanton, CA). White light (brightfield) and fluorescence image were obtained. For GFP, a 469/35 nm band pass excitation filter and a 525/50 nm band pass emission filter were used. For Tomato Red and RFP the excitation filter was 562/40 nm and the emission filter was 624/40 nm.

### Histology

Eyes were preserved in Davidson fixative and paraffin embedded overnight. Six μm sections were made through the optic nerve, deparaffinized and stained with hematoxylin and eosin (H&E). For immunofluorescence on sections, mice were anesthetized and transcardially perfused with 4% paraformaldehyde. Eyes and optic nerves were collected and cryoprotected with 30% sucrose. The tissue was embedded in Tissue Tek OCT compound followed by freezing in an isopentane bath cooled by liquid nitrogen. To reduce non-specific binding, 10–40 μm thick sections were blocked with 10% normal donkey serum for 1 h followed by staining for CD11b (BDPharmingen), CD11c-GFP (Invitrogen), Ki67 (Abcam), SMI-31 (Covance) and IsolectinB_4_ (Invitrogen). Sections were incubated as described [54, 62] with antibodies overnight at 4 °C. After three rinses in PBS, appropriate secondary antibodies were applied (Alexa Fluor 350, 488 and 594; Molecular Probes, CA). The tissues were incubated at RT in the dark for 3 h and counterstained with DAPI. Primary antibody was omitted to confirm specificity. Fluorescence images were obtained using a confocal scanning laser microscope (Olympus Fluoview 1000) or a LEICA DM 4000B fluorescence microscope.

### Parabiosis

B6J and ACTb^GFP^ mice were surgically conjoined at three months of age using the procedure of Kamran et al. [28]. From two to six months later, blood was taken from each parabiont to confirm successful parabiosis, followed by a unilateral ONC, or were untreated, to explore recruitment of GFP^+^ mononuclear cells into the B6J mice from the circulation by a retinal injury. At the specified times, the mice were perfused and separated for tissue harvest and analysis.

### RGC counts

RGC counts were done as previously reported [19]. Injection of 4% Fluorogold (FG) into the superior colliculus was used to label the RGC for counting. FG was administered after the ONC, 4 days before retina harvest. Labeled soma were counted by fluorescence microscopy.

### Statistical analysis

Analysis was done by one-way ANOVA with Tukey HSD post-hoc analysis or by Kruskal-Wallis H test analysis. *P* value of < 0.05 was considered significant.

### Ethics approval

All experiments conformed with the Association for Research in Vision and Ophthalmology Statement for the Use of Animals in Ophthalmic and Vision Research. Experimental procedures were approved by the University of Minnesota Institutional Animal Care and Use Committee.

## Results

### CD11c-GFP^hi^ myeloid cell response to an optic nerve injury

Studies of the origin and frequency of GFP^hi^ cells in the retina of CD11c^GFP^ mice after an ON injury required reproducible injuries. A well-controlled, limited ONC using DSAEK forceps [19] resulted in the stable loss of approximately 50% of the RGC, corresponding to loss of 25 × 10^3^ RGC/retina (Fig. 1a), a small fraction of the 8 × 10^6^ neurons/retina [25]. A consistent feature of the early GFP^hi^ cell response to this optic nerve injury was the accumulation of GFP^hi^ myeloid cells around the optic disk detected by 6 days post-injury (Fig. 1b). Microglia and the GFP^hi^ cells were readily distinguished by flow cytometry of retinas gated on CD45^med^CD11b^+^Ly6G^−^ cells from CX3CR1^YFP-creER^:CD11c^GFP^ double transgenic mice (Fig. 1c). The GFP^hi^YFP^hi^ subset increased dramatically post-ONC, peaking between 7 and 13 days post-ONC, while the GFP^lo^YFP^hi^ microglia showed a smaller increase (Fig. 1d).

### Parabiosis showed that increased retinal mononuclear cell numbers post-ONC was not due to recruitment from the circulation

**Fig. 2:**
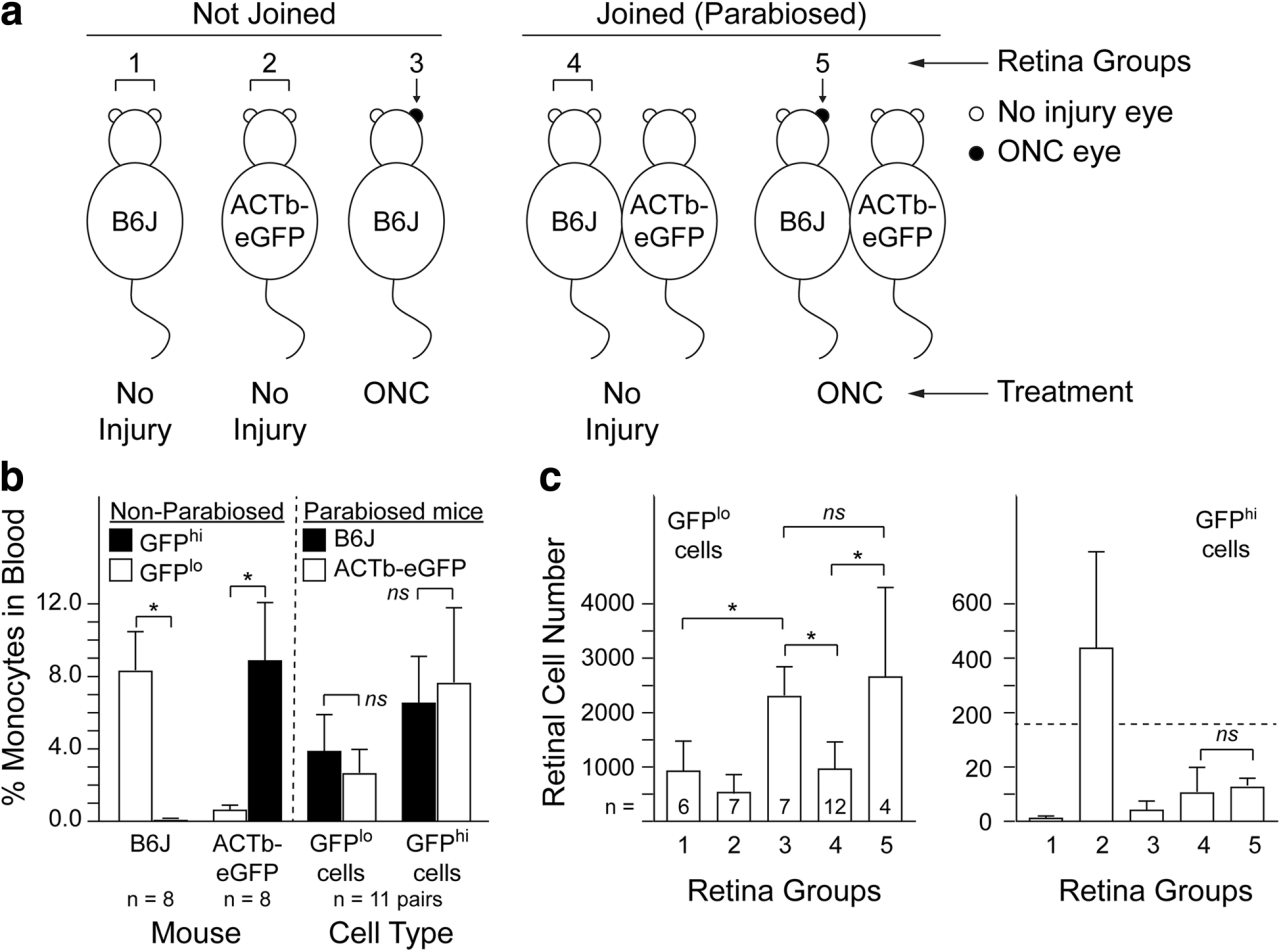
Parabiosis shows that the mononuclear cell response within a retina to an ONC is not mediated by recruited, circulating macrophages. **a** Experimental design of the parabiotic mice and controls. Retina groups and treatment are indicated. For non-injured mice (no ONC; groups 1, 2, and 4), both retinas from individual mice constituted individual samples. For ONC mice (groups 3 and 5), only the retinas from injured eyes (black filled) constituted samples. **b** Flow cytometry analysis of blood showing successful parabiosis. Circulating monocytes (CD45^+^CD11b^+^) were analyzed four to 6 months post-parabiosis for GFP. Results presented as GFP^lo^ or GFP^hi^ monocytes as a percent of all blood mononuclear cells. For parabiotic mice, both members of a pair were analyzed for GFP^lo^ and GFP^hi^ monocytes. **c** Flow cytometry analysis of retinal myeloid cells. Non-parabiotic mice were aged-matched to parabiotic mice. Mice were analyzed 7–9 days post-ONC. Results presented as number of GFP^lo^ or GFP^hi^ myeloid cells (CD45^med^CD11b^+^Ly6G^−^) per retina. Statistics were done by ANOVA with Tukey HSD post-test with number of mice analyzed indicated, * = *p* value of < 0.05, ns = not significant

By joining the circulations of two mice (parabiosis) in which one mouse carries readily detectable, labeled immune cells, it is possible to determine if those immune cells in tissues of the non-labeled mouse were derived from the circulation. Here, we used this approach to determine if ONC injury can stimulated the recruitment of circulating monocytes cells to the retina. Parabiotic pairs were made by joining *wt* B6J mice with ACTb^eGFP^ mice (Fig. 2a). Establishment of parabiosis was verified by flow cytometry analysis of blood monocytes (CD45^+^CD11b^+^) for GFP from each parabiotic partner four to six months post-joining and from unpaired B6J and ACTb^eGFP^ control mice (Fig. 2b). GFP^hi^ monocytes were rare in the blood of normal B6J mice but dominant in ACTb^eGFP^ mice (Fig. 2b, left). In contrast, blood from B6J x ACTb^eGFP^ parabionts revealed similar levels of GFP^hi^ and GFP^lo^ monocytes in either partner (Fig. 2b, right). To confirm that mononuclear cells from an ACTb^eGFP^ donor populating a B6J recipient could respond to stimulus, a needle stick injury was made in the brain of the B6J partner of a B6J x ACTb^eGFP^ parabiotic pair. Fluorescence microscopy of the injury site showed it was heavily infiltrated by ACTb^eGFP+^ cells (data not shown).

Flow cytometry of isolated retinas from unpaired control mice showed the background level of GFP^hi^ cells in B6J mice to be very low (retina group 1), whereas control ACTb^eGFP^ mice contained substantial numbers of GFP^hi^ cells (retina group 2) (Fig. 2c, right). The ability of retinal myeloid cells to respond to an ONC injury was confirmed in the B6J mice (Fig. 2c, GFP^lo^ cells in retina group 3 versus retina group 1). Following confirmation of successful parabiosis, myeloid cells in the retinas of the B6J partners in B6J x ACTb^eGFP^ parabionts were analyzed. Very few GFP^hi^ myeloid cells were found in retinas of uninjured B6J partners (retina group 4). ONC to the B6J partners increased the total retinal myeloid cell numbers in the B6J partner mice to a similar level observed in unpaired B6J mice given an ONC (Fig. 2c, GFP^lo^ cells, retina group 3 vs group 5). However, the number of GFP^hi^ myeloid cells was not increased by ONC to one eye of the B6J partners (Fig. 2c, right, retina group 4 vs. group 5). The initial flow cytometry analysis of the B6J partner mice yielded very low numbers of GFP^hi^ cells. To verify this low frequency of GFP^hi^ cells in the B6J partners, three additional retinas from uninjured B6J partners and two additional retinas from ONC injured B6J partners were analyzed by fluorescence microscopy for GFP^+^ cells. Uninjured retinas from the B6J partners contained a total of one to three GFP^+^ myeloid while the injured retinas contained three and five GFP^+^ myeloid (data not shown), thus confirming the low GFP^hi^ cell number observed by flow cytometry. Together, this data suggests that while the blood of the B6J partners was well-populated with ACTb^eGFP+^ cells, they did not contribute to the large increase in myeloid cells in B6J retinas post-ONC.

### Fate mapping the CD11c-GFP^hi^ cells revealed their microglial origin

**Fig. 3:**
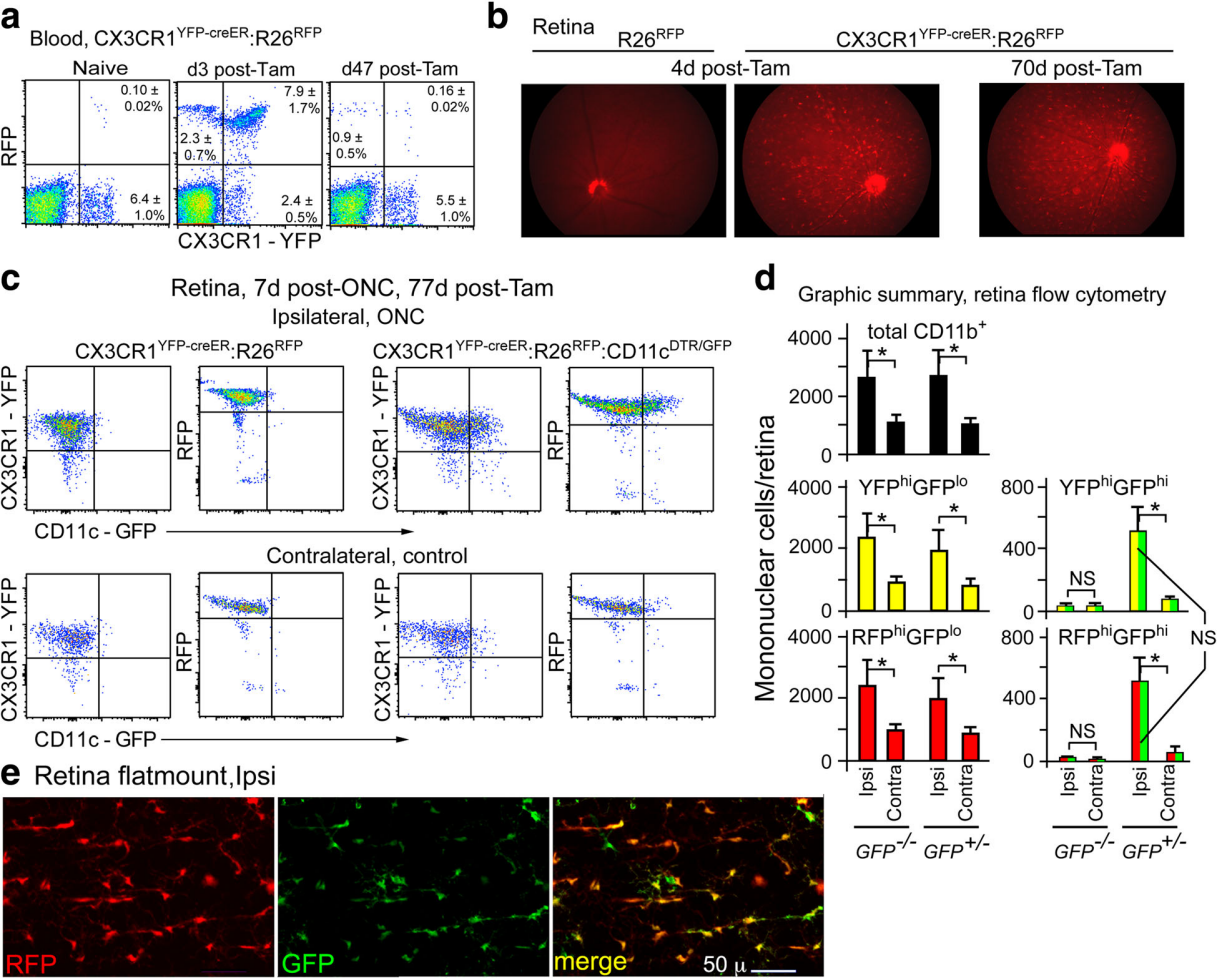
Fate mapping showed that the retinal GFP^hi^ myeloid cells post-ONC were derived from microglia. **a** Tam induction of the RFP reporter in CX3CR1^YFP-CreER^:R26^RFP^ mice labeled circulating CD11b^+^CD45^hi^ mononuclear cells, which substantially declined by 47 days. **b** Fundus photography showed that the retinal microglia were well labeled and expressed RFP at 70 days. **c** Flow cytometry showed that the microglia were induced to stably express RFP. (Left panels) An ONC-injured retina (ipsilateral) in mice lacking the CD11c-GFP transgene responded 7 days later with an increased number of microglia (YFP^hi^RFP^hi^) relative to the control (contralateral) retina. (Right panels) CX3CR1^YFP-CreER^:R26^RFP^:CD11c^GFP^ mice upregulated GFP on the YFP^hi^RFP^hi^ microglia in the ipsilateral ONC retinas, accounting for a substantial portion of the increase in retinal myeloid cells. **d** Quantitation of the flow cytometry from **c** showing averaged results of groups of 3 to 6 mice. All GFP^hi^ cells were also RFP^hi^ and YFP^hi^. Abbreviations: GFP^−/−^ = CX3CR1^YFP-CreER^:R26^RFP^ mice. GFP^+/−^ = CX3CR1^YFP-CreER^:R26^RFP^:CD11c^GFP^ mice. Flow cytometry on retina included gating for viability, doublet exclusion and FSC/SSC prior to gating on CD45^med^CD11b^+^Ly6G^−^ for expression of RFP, YFP and GFP. **e** Flatmount focused on the NFL of a retina post-ONC in a Tam-induced CX3CR1^YFP-CreER^:R26^RFP^:CD11c^GFP^ mouse showed that the GFP^hi^ cells were RFP^hi^

If the myeloid cells responding to an ONC injury were not recruited from the circulation, we asked if they had a local origin. In particular, did they arise from the resident microglia. CX3CR1^YFP-CreER^:R26^RFP^ mice, with and without the CD11c^GFP^ transgene, were systemically treated with Tam to induce expression of the floxed RFP reporter in all CX3CR1^+^ cells. RFP was highly expressed in circulating mononuclear cells 3 days post-Tam and in retinal microglia (Fig. 3a & b). By 47 days the blood was nearly cleared of RFP^hi^ cells (Fig. 3a), but retinas remained highly labeled at 70 days (Fig. 3b). An ONC was done at 70 days post-Tam followed by harvest at 78 days for flow cytometry and retinal flatmounts. The numbers of CD11b^+^CX3CR1^+^RFP^+^ cells with and without the CD11c^GFP^ reporter in the injured, ipsilateral retinas were indistinguishable (Fig. 3c & d), showing that the increase in myeloid cells in ipsilateral retinas was of the same magnitude in mice with or without the CD11c^GFP^ reporter. However, much of the increase in post-ONC myeloid cells was found to be due to the increase in the GFP^hi^ population, which was both YFP^+^ and RFP^+^ (Fig. 3d, right panels). Since recruited cells would have been RFP^−^, we concluded that GFP expression from the CD11c^GFP^ promoter/reporter marked the responding subpopulation of RFP^+^ microglia. No evidence of recruited cells (RFP^lo^GFP^hi^) was found. A flatmount of a Tam-induced, ONC-injured retina from an RFP^+^ reporter mouse showed that GFP^hi^ microglia were also RFP^hi^ (Fig. 3e).

### Extent of optic nerve transection affected the topography of the GFP^hi^ microglia response in retina

In preliminary studies we observed a vigorous injury response of GFP^hi^ microglia in the optic nerve post-ONC (Additional file 1: Figure S1). This observation raised the possibility that this response could contribute to the retinal ONC response. If the damaged axons in the optic nerve acted as an attractant and/or path for microglial migration into retina, then a full transection injury to the RGC axons in the optic nerve, while sparing the ophthalmic artery, might reduce the overall response in the retina by blocking the pathway of microglia migration from the optic nerve into the retina. Conversely, a partial ONT that also spared the ophthalmic artery might support a strong retinal response by leaving part of the pathway intact for migration. ONT procedures led to loss of RGC, but the retina was otherwise intact (Additional file 2: Figure S2b). Accidental transection of the ophthalmic artery during the optic nerve transection surgery rapidly produced a catastrophic, hypoxic injury to the retina (Additional file 2: Figure S2b); these samples were omitted. An ONT that spared the ophthalmic artery was found to be a potent stimulus for a microglial response in the retina (Additional file 2: Figure S2d). Accordingly, we sought to determine if a full vs partial ONT could be used to test our hypothesis if the ophthalmic artery was intact.

**Fig. 4:**
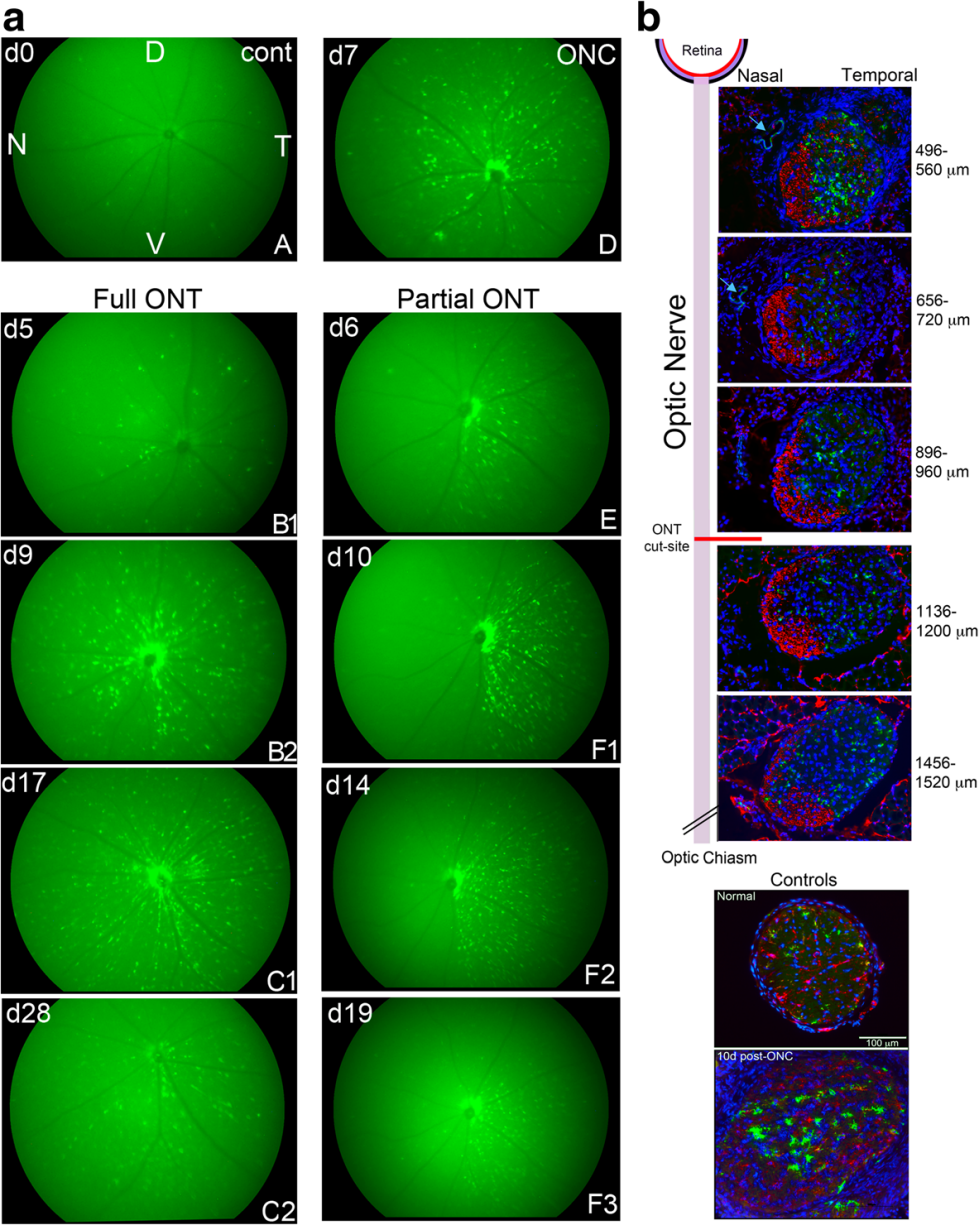
Fluorescence imaging of the fundus and optic nerve demonstrated the topography of axon loss and response by GFP^hi^ cells. **a** GFP-fluorescence fundus imaging was done post-ONT to monitor the progression and location of GFP^hi^ cells in the retinas from the left eye of CD11c^GFP^ mice. Mouse A is an untreated control and shows the retinal orientation in all fundus photos (D-dorsal, V-ventral, N-nasal, T-temporal). Mouse D is 7 days post-ONC. Fundus photos of mouse B (5 and 9 days post-full ONT) and mouse C (17 and 28 days post-full ONT) show the progression of GFP^hi^ cells in retina. The response to a partial ONT is shown in the panels of mouse E and F. The panels are similarly labelled and dated for days post-ON injury. Only GFP^hi^ cells are visible. **b** Cross sections of the optic nerve post-injury in CD11c^GFP^ mice. The distribution of anti-SMI-31 staining for RGC axons late at 35 days post-partial ONT correlated with the extent of the transection (two-thirds cut) estimated from fundoscopy. Blue = DAPI; Green = CD11c^GFP^; Red = SMI-31; Light blue = Isolectin B_4_. Bottom panels show control and 10 days post-ONC. Blue = DAPI; Green = CD11c^GFP^; Red = anti-CD11b

To assess the potential contributions of the optic nerve to the retinal microglia response due to partial cut vs full cut ONT, it was helpful to determine the extent of the ONT prior to harvesting the retina for analysis. We previously showed that the GFP^hi^ cells preferentially associated with the RGC axons after an ONC, generating a radial pattern in fluorescence microscopy and fundus imaging [19, 33]. Due to the topography of the RGC axons projecting into the optic nerve during development, we predicted that a partial transection of the optic nerve would lead to a limited sectoral association of GFP^hi^ microglia on the axons that were severed in the ON, while a full transection would give a 360° pattern of GFP^hi^ microglia. Conversely, an ONC would yield no specific sectoral pattern. These predictions were verified by fundus imaging (Fig. 4a). Very few GFP^hi^ microglia were found in normal CD11c^GFP^ retina (panel A), but were prominent throughout the fundus 7 days post-ONC with no discernable sectoral distribution (panel D). Cuts intended to result in a full transection gave the fundus images in panels (B1 to C2), where the GFP^hi^ microglia are found in all sectors. Fundus imaging of attempts to make partial ONTs on the temporal side of the optic nerve led to a reproducible, sectoral appearance of GFP^hi^ cells in approximately one-third to one-half of the retina (panels E to F3).

To validate the use of fundus imaging to detect axon topography, an ipsilateral optic nerve from a mouse 35 days post-partial ONT was severed flush with the back of the globe. The other end was cut free at the chiasm. The nerve was prepared for cross-sections and stained for SMI-31 to detect axons, and for isolectin B_4_ to detect the ophthalmic artery, providing orientation. The sectoral distribution of GFP^hi^ microglia seen in retina was recapitulated in the optic nerve receiving a partial transection (Fig. 4b). The transection procedure cuts from the temporal side behind the globe so that the ophthalmic artery was on the opposite, nasal side of the nerve. Accordingly, the SMI-31 staining (red) of intact fibers was concentrated on the nasal side of the nerve, while GFP^hi^ cells were concentrated on the temporal side, where the SMI-31 stainable axons have been substantially cleared. Controls showed the density of CD11b^+^ and GFP^hi^ cells in normal and 10 days post-ONC controls.

### Time course of post-injury changes in the appearance and distribution of GFP^hi^ and GFP^lo^ microglia in the retinal NFL/ganglion cell layer

**Fig. 5:**
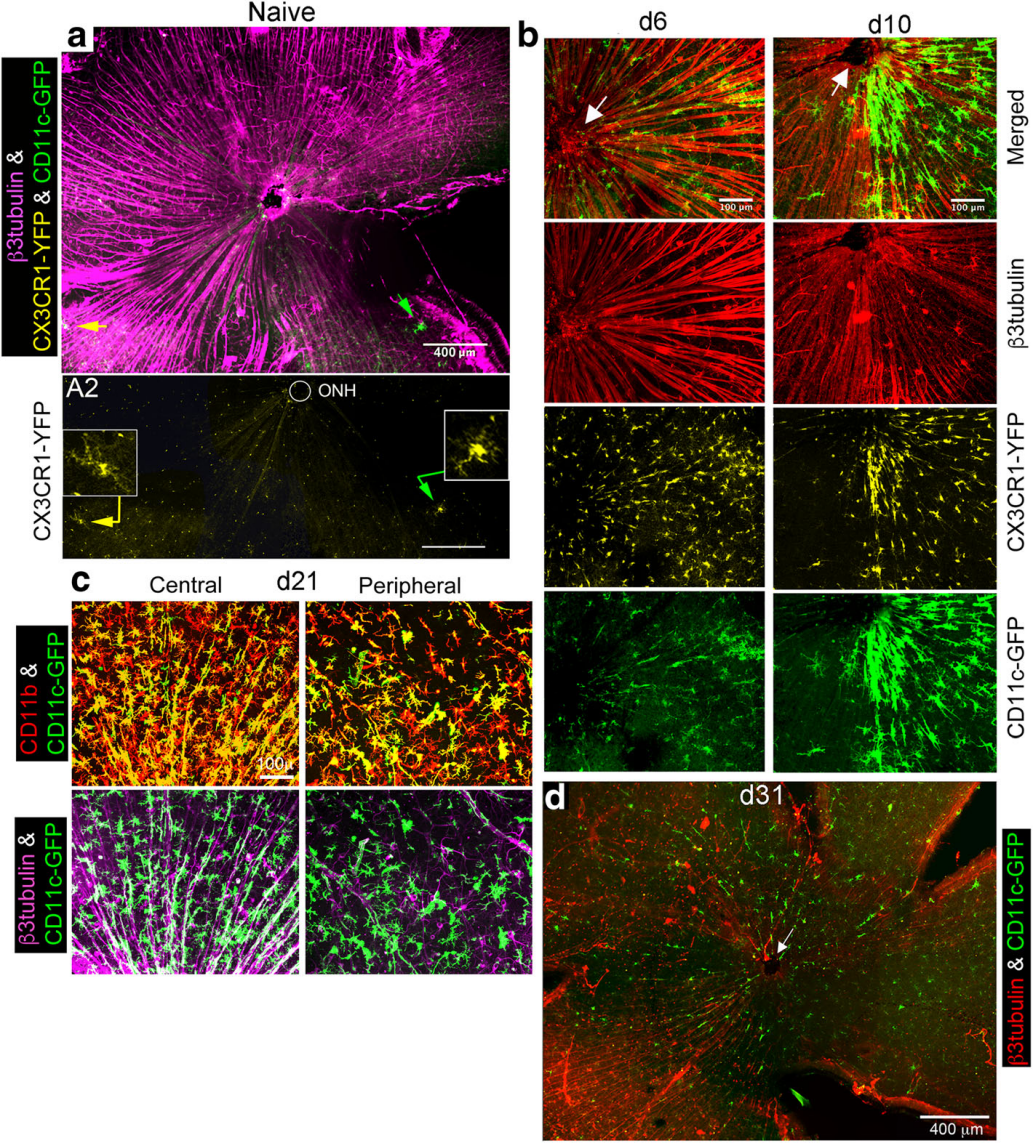
Presence and distribution and of GFP^hi^ and YFP^hi^ microglia in naïve retina and at days 6, 10, 21, and 31 post-ONT in CX3CR1^YFP^:CD11c^GFP^ mice. **a** Confocal stack of the RGC/NFL in naive retina showing the ONH and peripapillary region. Yellow and green arrows point to a YFPhiGFPlo cell and a YFPhiGFPhi cell, respectively. (A2) Enlarged view (insets) of the two ramified microglia shown above. Visualization of the YFP channel only results in both cells appearing yellow in this image. **b** Appearance of GFP^hi^ cells in the ipsilateral retinas 6 and 10 days after a partial ONT showing the rapid progression of the GFP^hi^ cell response. **Note**: The day 10 retina is the same retina as shown in panel E day 6 fundus photo in Fig. 4**a** of partial transections. Red = β3-tubulin; Yellow = YFP; Green = GFP. White arrows point to the ONH. **c** Dense association of GFP^hi^ cells with nerve fibers in the ipsilateral retina at 21 days post-partial transection. **d** GFP^hi^ cells declined substantially in retina by 31 days post-ONT

Close examination of naive retinal flatmounts found few YFP^hi^ microglia near the optic nerve head in a confocal stack containing only the NFL and RGC layers (Fig. 5a). The microglia marked by arrows in panel A were enlarged to show that they were YFP^hi^GFP^lo^ and YFP^hi^GFP^hi^, respectively panel A2. Many more microglia were found in the underlying inner plexiform layer (IPL) (Additional file 3: Figure S3). The findings seen in the fluorescence fundus imaging were further recapitulated in retinal flat mounts. GFP^hi^ cells were found in the early stages of aligning with the nerve fibers in the ipsilateral retina at 6 days post-full ONT (Fig. 5b), while there was minimal change in GFP^hi^ cells in the contralateral retina (Additional file 4: Figure S4a). By 10 days post-partial ONT there was pronounced association of GFP^hi^ cells with the nerve fibers near the ONH on the temporal half of the retina (Fig. 5b). GFP^hi^ cells also appeared in peripheral retina along the nerve fibers (Additional file 5: Figure S5). The background level of GFP^hi^ cells in the contralateral retina appeared to have increased (Fig. 5b), but may represent intraretinal migration since the total number of cells/contralateral retina were minimally elevated, and association with the NFL was minimal.

At 21 days post-partial ONT an intense interaction of GFP^hi^ microglia with RGC axons was seen in the ipsilateral retina, in both central and peripheral retina (Fig. 5c). GFP^hi^ microglia appeared as green and yellow cells due to CD11b co-staining using a red fluor. In the contralateral retina, an increase in GFP^hi^ microglia in the peripapillary region was seen (Additional file 4: Figure S4b) compared to naïve retina, and 6 and 10 days post-ONT. The important difference was that the GFP^hi^ microglia in contralateral central retina were not closely associated with axons but were ramified (Additional file 4: Figure S4b). Similar differences were found in the interactions between GFP^hi^ microglia in the ipsi- versus contralateral peripheral retina. The number of GFP^hi^ cells in peripapillary retina after a near-complete ONT declined substantially by 31 days (Fig. 5d), and the staining for RGC axons with anti-β3 tubulin was sparse compared to normal retina, and limited to the lower-left quadrant, consistent with the topography of a substantial ONT.

### Quantitation of GFP^hi^ and GFP^lo^ cells in the layers of naive and post-ONC CD11c^GFP^ retina

**Fig. 6:**
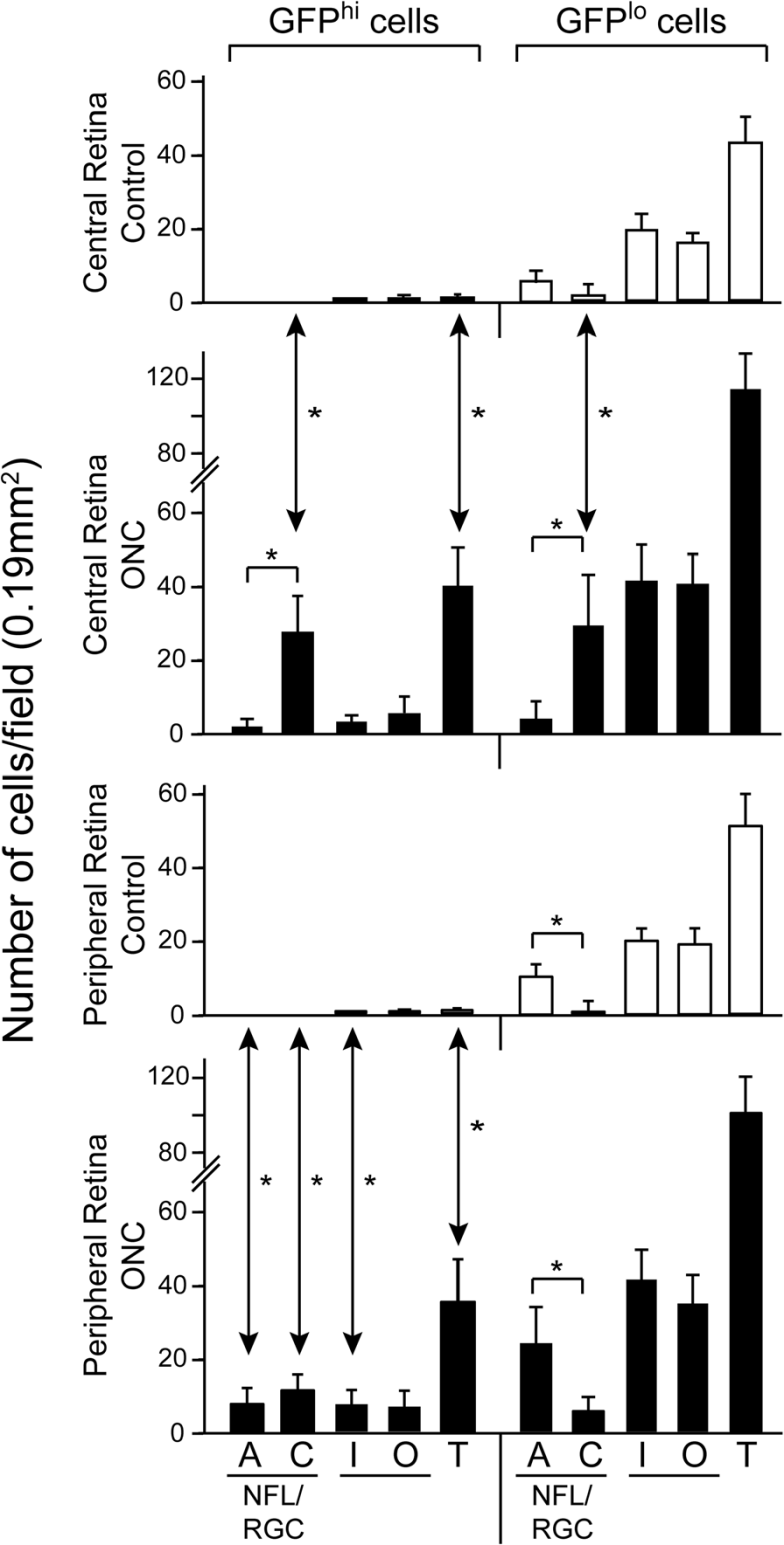
The distribution of GFP^hi^ and GFP^lo^ microglia in the naive retina and 10 days post-ONC in CD11c^GFP^ mice. Cells in the NFL/RGC were divided into two categories, those adjacent (A) to nerve fibers or RGC soma and those in contact (C) with nerve fibers or RGC soma (see, Additional file 6: Figure S6). Cells in the IPL (I) and OPL (O) were counted, and total numbers (T) are given. Each bar represents the average of 4 retinas with 4 fields in each retina, whether central or peripheral, and repeated for each of the 4 layers. *, *p* < 0.05

Although flow cytometry measurements of cell number in the retina show differences in the overall magnitude of the responses to optic nerve injury, and photomicrographs of flatmounts show direct evidence of the interactions between the myeloid cells and injured neurons, counting cells can show changes in specific sites. Retinal flatmounts of naive and day 10 post-ONC retinas were made. In addition to counting the IPL and OPL which contain significant populations of microglia in control and injured retina, the NFL/RGC was examined for GFP^hi^ and GFP^lo^ microglia in two configurations. Each cell was examined for GFP expression, and for its association with nerve fibers or RGC soma, whether adjacent to the fibers or soma (Additional file 6: Figure S6a), or in direct contact (Additional file 6: Figure S6b). Care was taken to avoid contaminating the NFL/RGC counts with cells in the IPL, as the IPL is always well-populated with microglia. Eight areas of each retina were counted (Additional file 6: Figure S6c); the results of the four central areas were combined, according to the layers and interactions in the NFL/RGC, and whether GFP^hi^ or GFP^lo^, as were those of the four peripheral areas. The largest differences between counts of injured and control retina were the GFP^hi^ and GFP^lo^ cells found in direct contact in the central NFL/RGC (Fig. 6). This was consistent with the results seen on days 6 through 21 post-injury shown in Fig. 5. In peripheral retina the GFP^hi^ cells were elevated in every category post-ONC. GFP^lo^ cells in the IPL and OPL essentially doubled in both central and peripheral retina post-ONC.

### The microglia response to injury in the CX3CR1^YFP^:CD11c^GFP^ optic nerve is robust

**Fig. 7:**
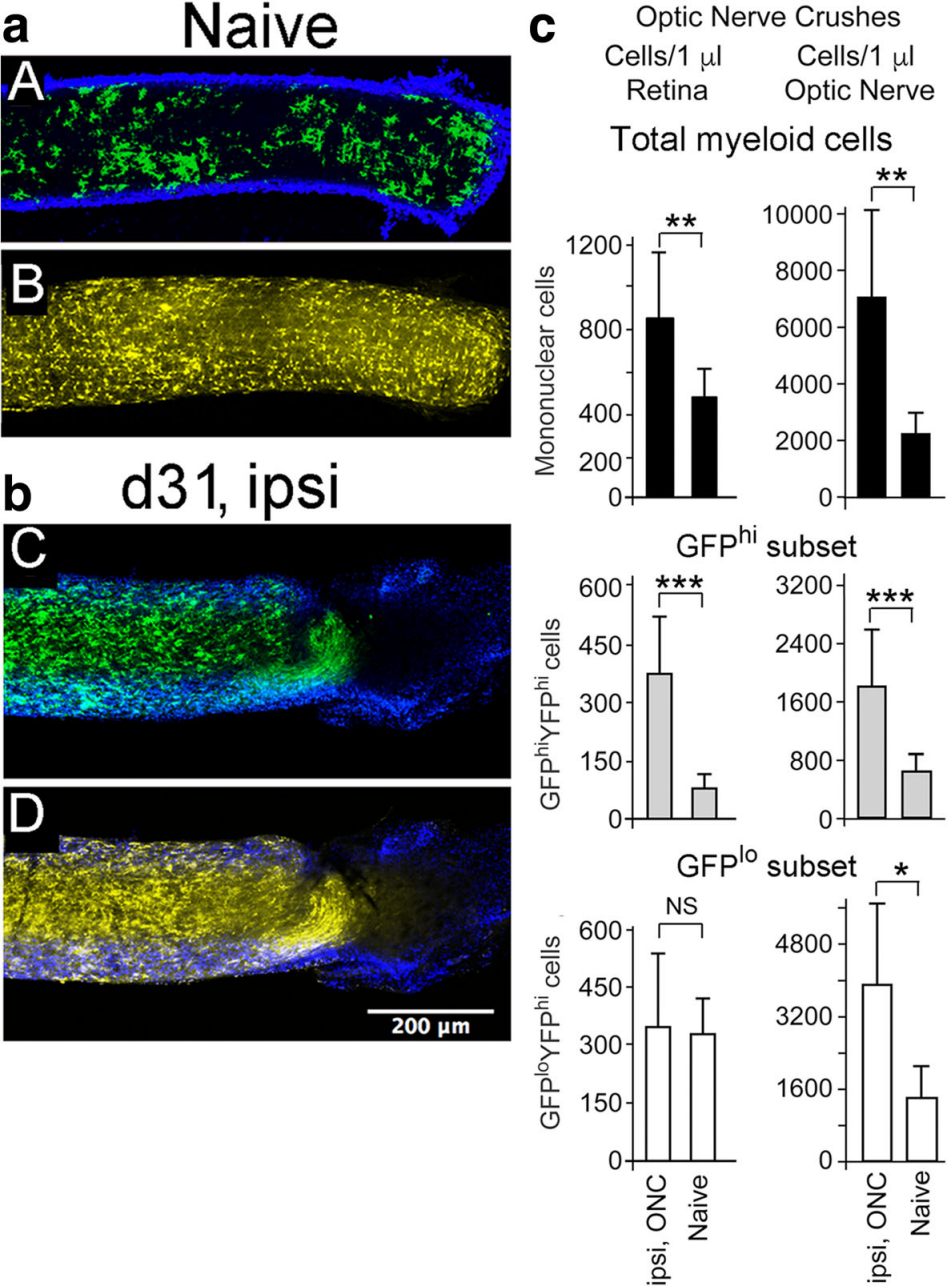
Naive optic nerve of the CX3CR1^YFP-creER^:CD11c^GFP^ mouse was well-populated with microglia and generated a strong response to injury. **a** GFP^hi^ microglia were numerous in naïve optic nerve (A), and GFP^lo^ microglia densely populated the same naïve optic nerve as shown in (B). The Z-stack of flat mounted optic nerve shown in panels A and B was obtained by scanning through the optic nerve to find its center, and then stacking six 3 μm optical sections centered in the middle of the nerve. **b** The optic nerve at 31 days post-ONC remained densely populated. CD11c^GFP^ reporter = green; DAPI = blue; CX3CR1^YFP^ reporter = yellow. **c** Quantitation of retinal myeloid cells by flow cytometry of retina and optic nerve from naïve and day 10 post-ONC donors. An optic nerve crush led to increases in GFP^hi^ and GFP^lo^ microglia in retina and optic nerve. Cells were gated on viable CD45^med^CD11b^hi^Ly6G^−^ cells with doublet exclusion. GFP^hi^ and GFP^lo^ cell counts were limited to CX3CR1-YFP^hi^ cells. Mean ± SD. NS, not significant; *, *p* < 0.05; **, *p* < 0.01; ***, *p* < 0.001. Statistical analysis by one-way ANOVA with Tukey HSD post-test. 6–11 mice/group

The same nerve fibers that attracted little myeloid cell presence in the naïve retina were densely surrounded by GFP^lo^ microglia in the naive optic nerve, which was also well-populated with GFP^hi^ microglia (Fig. 7a). At 31 days post-ONC the optic nerve remained highly populated with GFP^hi^ and GFP^lo^ microglia (Fig. 7b). Very few GFP^hi^ or GFP^lo^ microglia were found in the optic nerve sheath. Analysis of the myeloid cells in the optic nerve and its injury response was compared to that of the retina. The average volume of an isolated optic nerve based on a diameter of 249 ± 43 μm and a length of about 5 mm from the eye to the optic chiasm was approximately 0.24 mm^3^, far less than the 2.73 mm^3^ volume of a typical single retina. To compensate for the differences in volume of the optic nerve compared to retina, the assays were expressed as the number of myeloid cells/1 μl of tissue. The recovery of total CD45^med^CD11b^hi^ cells from naive optic nerve was much greater, approximately 5-fold, than that from naive retina on a volume basis, approximately 2.2 × 10^3^ cells/mm^3^ for the optic nerve compared to 0.48 × 10^3^/mm^3^ for the retina. The density of GFP^lo^ microglia in naive optic nerve was several fold higher than in retina, and this difference was magnified 10 days post-ONC (Fig. 7c). GFP^hi^ cells were also much higher in naive optic nerve, and in post-ONC samples. The differences between the GFP^lo^ and GFP^hi^ populations of microglia in naïve tissue were similarly pronounced. An important difference between retina and optic nerve post-ONC is that the GFP^lo^ microglia in retina were not significantly increased after the ONC, while the difference in the optic nerve pre- vs post-ONC was substantial (Fig. 7c, bottom panels).

### A full ONT limits the retinal response to the optic nerve injury

**Fig. 8:**
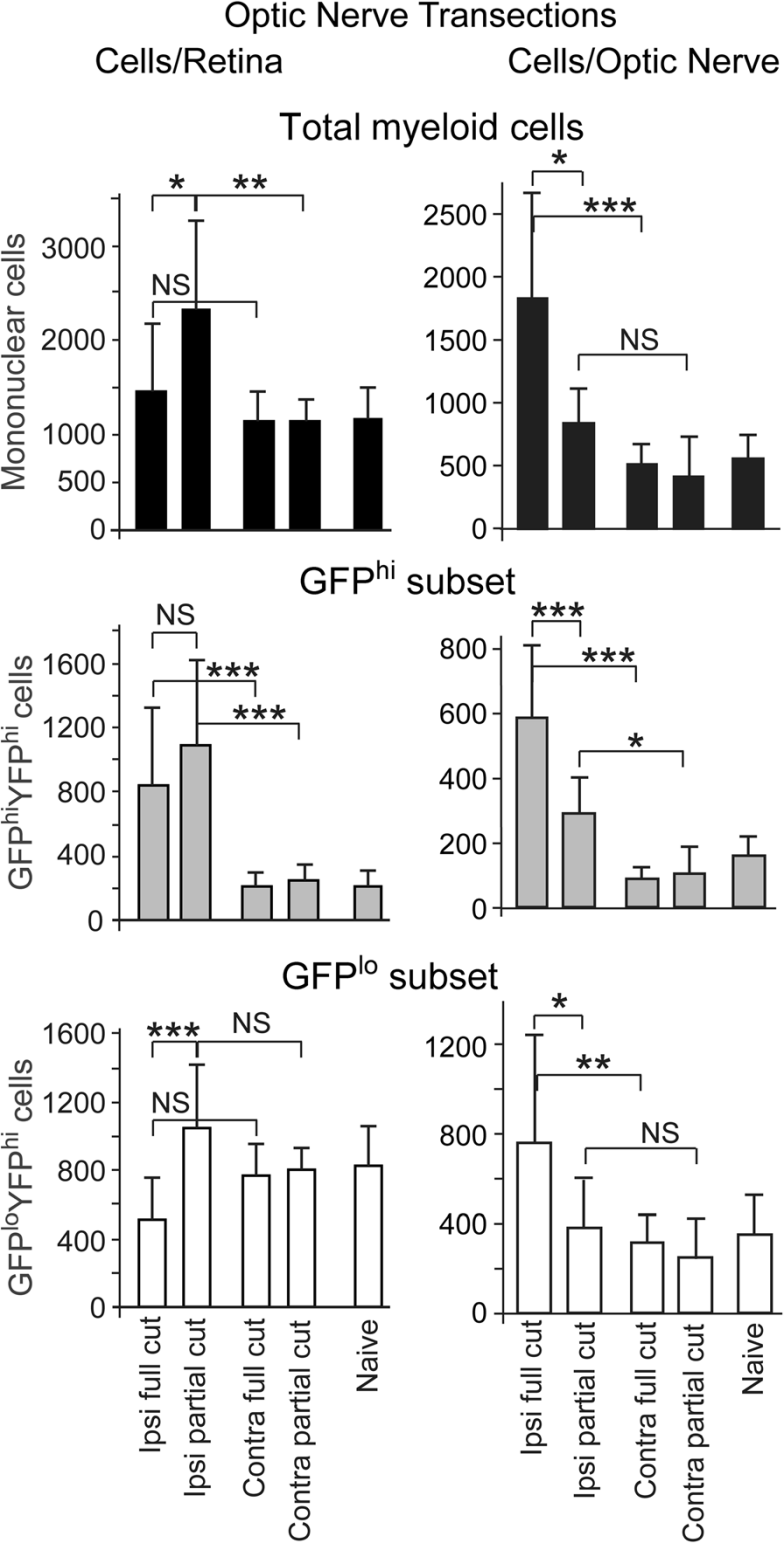
Full thickness ONT limited the magnitude of the retinal myeloid cell response in comparison to a partial nerve transection. The ophthalmic artery was spared in all transections. Retinas and optic nerve were harvested from CX3CR1^YFP-creER^:CD11c^GFP^ mice at 11 days post-injury. The volume of retina was approximately 10-fold greater than optic nerve. The full length of the optic nerve was recovered for flow cytometric analysis. GFP^hi^ and GFP^lo^ microglia were gated on viable cells, doublet exclusion, expression of CD45^med^CD11b^hi^ and exclusion of Ly6G^+^ cells. GFP^hi^ and GFP^lo^ subsets were limited to CX3CR1^+^ cells. Mean ± SD. NS, not significant; *, p < 0.05; **, p < 0.01; ***, p < 0.001. Statistical analysis by one-way ANOVA with Tukey HSD post-test. 7–12 mice/group

The impact of partial and full ONT injuries on the recruitment of GFP^hi^ myeloid cells and GFP^lo^ microglia into the retina was done by comparing the results of the transections on day 11 post-injury in CX3CR1^YFP^:CD11c^GFP^ mice. Retina and optic nerve were assessed by flow cytometry. Based on the fundus photos of retinas acquired from live mice at days 9 to day 11 post-ONT, the retinas and nerves were assigned to the full or partial cut categories as demonstrated by the samples shown in Fig. 4. This grouping revealed differences in the outcomes of full- versus partial-ONT (Fig. 8). Full thickness ONT limited the magnitude of the total retinal mononuclear cell response in comparison to partial ONT; the GFP^hi^ cells in retina were elevated while the GFP^lo^ microglia numbers were lower than in the partial ONT. While the full ONT is the more severe injury, and accompanied by the loss of virtually all RGC in retina, the full ONT gave an attenuated GFP^hi^ microglia response relative to a partial ONT. Since the GFP^hi^ cells were not significantly lower in full ONT retinas compared to partial ONTs, it appears that a greater fraction of retinal microglia converted to GFP^hi^. Since retinal microglia were not replaced by migrants from the full cut optic nerve, their numbers were significantly lower than in the retina of a partial ONT (Fig. 8). The opposite results were found in optic nerve, where the full ONT samples were elevated compared to partial ONT samples.

### GFP^hi^ microglia in the optic nerve and optic nerve head

**Fig. 9:**
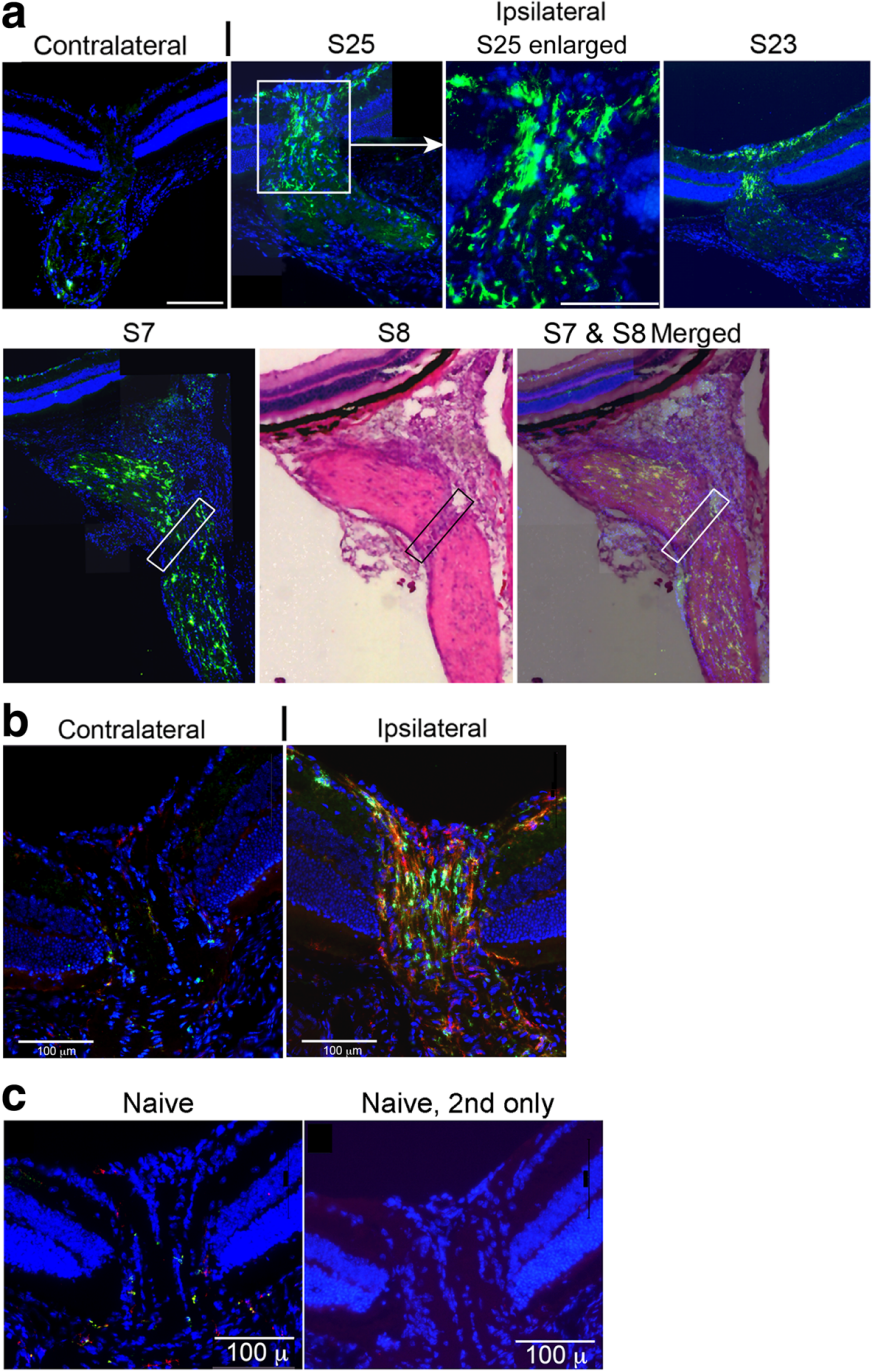
Comparison of CD11c-GFP^hi^ cells in the optic nerve and optic nerve head 11 days after an ONT. **a** Sections (S) prepared from ipsilateral eye following a partial ONT, and the contralateral retina/optic nerve. The site of the transection on S7 and S8 is indicated by the boxes on the stained and merged sections. The adjacent S7 section showing the GFP^hi^ cells was merged onto the H&E stained S8 section. **b** Sections prepared from an ipsilateral eye following a full ONT, and the contralateral eye. Sections included detection of GFP^hi^ cells and staining for CD11b on sections. **c** Naive retina and 2nd antibody only controls. Serial 12 μm thick sections were cut. White scale bar is 100 μm. GFP = green; anti-CD11b = red; DAPI = blue

The optic nerve head was nearly devoid of GFP^hi^ cells in naïve (Fig. 9c) and post-ONT contralateral retinas (Fig. 9a & b). In contrast, the ipsilateral optic nerve head was heavily populated with GFP^hi^ microglia at 11 days post-partial ONT (Fig. 9a, S25). S23 showed the extensive infiltration of GFP^hi^ microglia into the RGC/NFL around the optic nerve head that was prominent in views of flatmounts post-ON injury. GFP^hi^ cells were found on both sides of the partial cut site in the nerve (S7/S8). The ipsilateral ONH following a full ONT was heavily populated with GFP^hi^ and GFP^lo^ microglia (Fig. 9b).

### Optic nerve was populated with Ki67^+^ cells, but few were found in retina

**Fig. 10:**
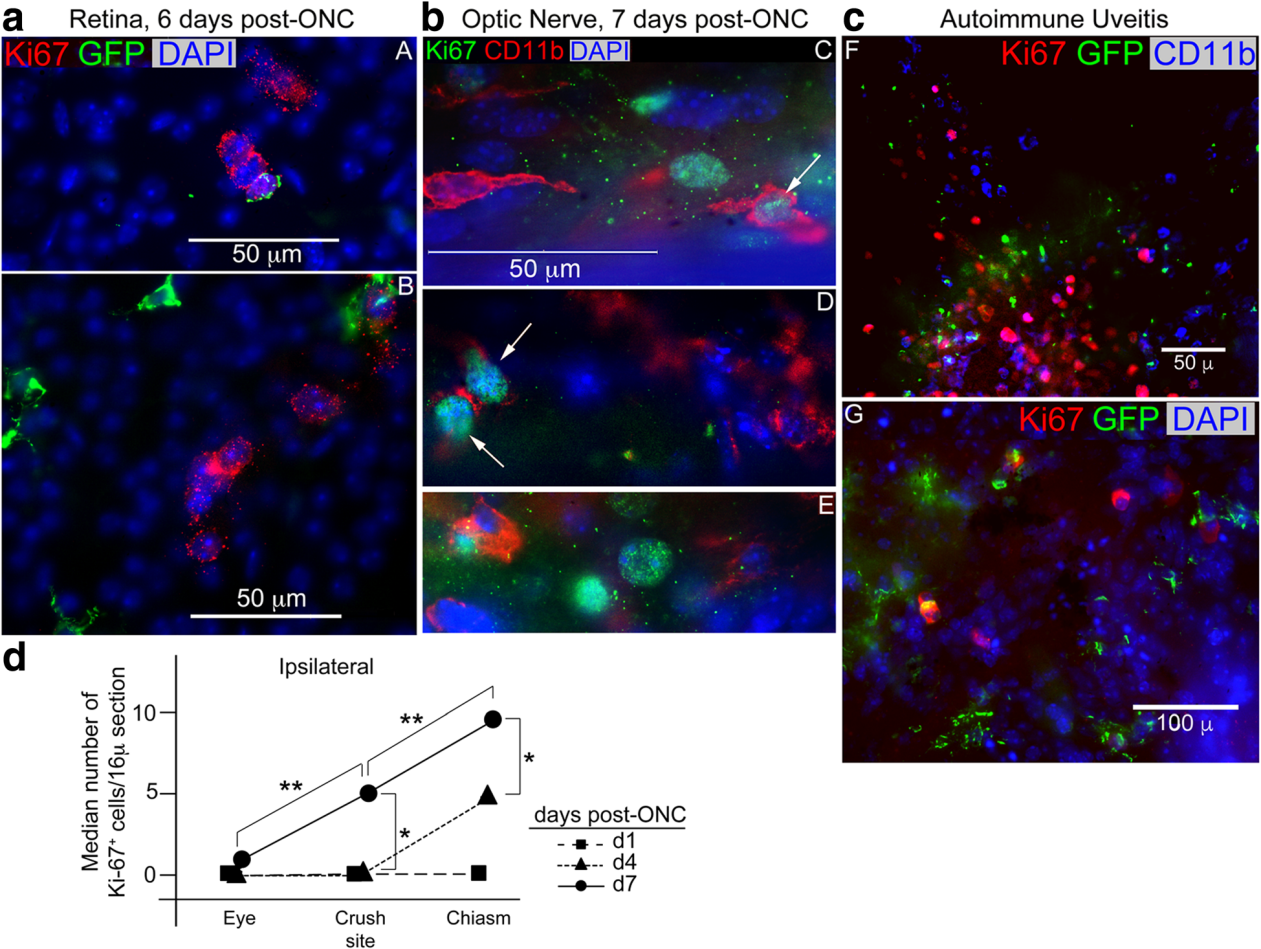
Detection of Ki67^+^ cells in the optic nerve and retina. **a** Rare Ki67^+^ cells found in retinal flatmounts of CD11c^GFP^ mice at 6 days post-ONC. **b** Analysis of the optic nerve of B6 mice 7 days post-ONC revealed Ki67^+^ cells; some were also CD11b^+^. **c** Onset of retinal inflammation in the R161 spontaneous murine model of autoimmune uveitis in R161H x CD11c^GFP^ mice on the (B10.R3 x B6)_F1_ background revealed extensive cell proliferation in retinal flatmounts prepared from eyes harvested at 111 days of age. (F) Many Ki67^+^ cells were found in the central retina near the optic nerve head, including some that co-stained with CD11b. (G) Smaller numbers of Ki67^+^ cells were found in the periphery of the opposite, inflamed retina. **d** Counts of Ki67^+^ cells in crushed ON. Sixteen micron thick sections were cut and stained. Non-parametric statistic done with Kruskal-Wallis H test analysis. *, p < 0.05; **, p < 0.01

Careful examination of retinal flatmounts from CD11c^GFP^ mice post-ONC or ONT at 2 or 6 days post-injury found very few Ki67^+^ cells**.** Quantitation of Ki67^+^ cells/retina was problematic in that setting up a counting grid of 8 regions, each 0.19 mm^2^, with 2 regions/petal, led to counts of 0 cells/retina. However, searching the entire retina for Ki67^+^ cells yielded approximately 1–3 small clusters or single cells/retina (Fig. 10a); some were GFP^+^, and some were CD11b^+^ (not shown). By comparison, many Ki67^+^ cells were found in retina using an inflammatory disease model, experimental autoimmune uveitis, that attracted large numbers of immune cells into retina (Fig. 10c). If the retina was not a source of new microglia, we asked if the optic nerve supported generation of the injury response. Unlike our observations in retina, Ki67^+^ cells with and without CD11b expression were readily found in the optic nerve after injury (Fig. 10b). Counts of Ki67^+^ cells in the optic nerve were made at 1, 4, and 7 days post-ONC (Fig. 10d) revealed substantial numbers of Ki67^+^ cells by 7 days post-ONC in the ipsilateral optic nerve. In contrast, very few Ki67^+^ cells were observed in the contralateral nerve (data not shown). The vigorous GFP^hi^ and GFP^lo^ microglia response in the optic nerve was well-placed to contribute to the cellular response found in retina via migration from the nerve into the retina. These results indicate that the higher number of myeloid cells, and especially GFP^hi^ microglia, in the retina after an ONC or partial ONT are not solely due to retinal microglial proliferation and activation in retina, but also represent migration from the nerve into the retina.

## Discussion

It has long been observed that injury of retinal ganglion cells leads to a response by innate immune cells, particularly retinal microglia [19, 26, 33, 37, 38, 65, 68], and that RGC apoptosis can result from even modest injury [2, 5, 10, 11, 41]. There is a substantial body of literature in which crush injuries or transection of the optic nerve has been used to model glaucoma, traumatic optic neuropathy, and CNS nerve regeneration [3, 11, 37, 39, 41, 48, 56]. Many of these studies have shown microglia to be associated with survival or clearance of injured axons [42, 43]. However, the precise mechanisms by which microglia contribute to axon survival/clearance and their role in neural modeling and regeneration are still a matter of study. Using the CD11c^GFP^ mouse we described a microglia-like population of cells uniquely identified by their expression of GFP in the retina after optic nerve injury that were distinct in response and function from other microglia. They differed in that they could act as dendritic cells, had a more dynamic response to injury, and were closely associated or in contact with the actual cells damaged by injury to the retina [18, 19, 33, 46]. Because of these unique characteristics, we sought to clarify the role and origin of these retinal GFP^hi^ myeloid cells. In this study we show that these cells were not derived from circulating macrophages but rather were generated from retinal GFP^lo^ microglia and augmented by microglial proliferation in the optic nerve.

Studies on the origin(s) of innate immune cells in the nervous tissue following injury have been both limited and complicated by the models used to examine the issue. Many studies have been done using radiation bone marrow chimeras. They showed there was a gradual replacement of myeloid cells in neural parenchyma by circulating macrophages. Other studies approached this issue by largely ablating resident microglia and examining their replacements. Both of these strategies tend to create niches that are biased towards the most rapid replacement and may recruit circulating macrophages. Indeed, with the ablation studies it has been noted that key growth and maintenance factors such as IL-34 and CSF-1 are produced, leading to the stimulation of CD115^+^ myeloid cell replacements to fill the niche. Conversely, parabiotic studies on the origins of myeloid cells in the brain following injury demonstrated that few responding cells were derived from the circulating macrophages [1, 31]. Since parabiotic studies can avoid the issue of artificially created, empty niches we found it useful in identifying the origin of retinal myeloid cells responding to injury or loss of axons. Similar to the results found in the brains of parabiosed mice, our results demonstrated that increased numbers of retinal myeloid cells after injury was not the result of cells being recruited from the circulation.

Since the enhanced appearance of myeloid cells in the retina after injury is not from a circulating source, it was logical to ask whether the GFP^hi^ cells in the retinas of CD11c^GFP^ mice after ONC injury were derived from the resident GFP^lo^ microglia. Our fate mapping experiments clearly demonstrated that the GFP^hi^ cells were indeed derived from GFP^lo^ microglia. Although referred to as GFP^hi^ retinal myeloid cells in this study, these cells can be thought of as GFP^hi^ microglia consistent with our previous studies showing these cells were CD45^med^CD11b^hi^CX3CR1^hi^Ly6G^−^Iba1^+^F4/80^+^Ly6C^lo^ [62].

If the development of GFP^hi^ retinal myeloid cells after injury was from the transition of GFP^lo^ retinal microglia, then it would be expected that the number of GFP^lo^ microglia in the retina after ONC injury would concomitantly decrease. However, the fact that we have consistently observed steady or transiently increased GFP^lo^ microglia numbers in the retina after ONC lead us to consider adjacent neural tissue as a source of these microglia. Since there are substantial numbers of microglia immediately underneath the NFL/RGC in the inner plexiform layer in naïve mice it could be expected that they could simply move up into the NFL/RGC following ONC. However, the course of the microglia response to an ONC begins with their appearance around the optic disk followed by a radial expansion through the peripheral retina. Interestingly, the optic nerve head itself in naïve mice has few microglia suggesting it is not the source of the responding GFP^hi^ and GFP^lo^ cells. This lead us to investigate whether the optic nerve could be a source of responding microglia. We hypothesized that if the optic nerve was responsible for generating responsive microglia that migrate into retina, then transection of the optic nerve close to the posterior pole, while sparing the artery, would limit access of optic nerve microglia to the retina. If this was the case, then a partial nerve transection should permit microglia access to the retina, but a full transection would block access to retina. Because transections were made from the temporal side, both the sheath and the nerve could be cut without damaging the artery. To facilitate the distinction between full and partial transections so that we could group the experimental mice accordingly, we took advantage of the topographic distribution of RGC axons in the proximal end of the optic nerve as described by others [34, 52], and the association of GFP^hi^ cells with injured nerve fibers in the retina. This provided us with a straight-forward fluorescence fundus imaging strategy which readily distinguished between partial and full transections. Fundus imaging of the optic nerve head and retina in CD11c^GFP^ mice showed that the area around the optic nerve head was highly populated with GFP^hi^ cells after an optic nerve injury, consistent with the potential for responding microglia to migrate along damaged nerve fibers in the optic nerve into the retina, contributing to the increase in retinal myeloid cells after injury. Except for the expected loss of RGC and their axons, the retinas were intact for the duration of the experiment. With respect to the loss of RGC and clearance of degenerating axons in the retina, the potency of the injury stimulus in the retina following a full ONT is more severe than our ONC protocol, since a full ONT severs all of the RGC axons in the optic nerve whereas an ONC leaves approximately 50% of the axons and the corresponding RGC to survive indefinitely [19]. Partial ONTs were also done to provide the essential control. Confocal studies of retinal flatmounts post-ONT showed GFP^hi^ cells associated with nerve fibers and RGC in the expected topography.

Flow cytometry of the retina after a full ONT versus a partial ONT showed that the microglia response in the retina differed. Despite the more severe injury to the RGC axons and subsequent apoptosis of the RGC in a full ONT, fewer total retinal microglia were found at 11 days post-full ONT compared to a partial ONT. Much of that difference in total microglia/retina was due to finding fewer GFP^lo^ microglia in retina after a full cut compared to a partial cut. Conversion of microglia to GFP^hi^ microglia depleted the GFP^lo^ microglia which could not be sustained by influx from the optic nerve due to the full cut ONT. Conversely, the total number of GFP^hi^ cells/retina was not significantly different between a full and partial transection. Closer examination of the data shows that a greater proportion of GFP^hi^ cells was generated from their retinal microglial origin in full transections; i.e. an average of 825 GFP^hi^ cells and 500 microglia following a full cut vs an average of 1100 GFP^hi^ cells and 1050 GFP^lo^ cells in the partial cut. The partial ONT led to a response that was similar in magnitude and composition to an ONC.

Analysis of the myeloid cell response in optic nerve showed differences from retina in quiescent and injured samples. The full transection gave significantly higher numbers of GFP^hi^, GFP^lo^, and total microglia in the nerve than found after partial transection. On the basis of cell number/unit volume the magnitude of the responses in the optic nerve was substantially higher than in retina given that the volume of the optic nerve is 10-fold less than that of the retina. The decreased response found in the GFP^lo^ population in retinas challenged by a full ONT is consistent with our hypothesis in that full transection may block migration of microglia from the optic nerve to the retina. Given the evidence that GFP^hi^ cells were derived from the GFP^lo^ microglia, we would predict a decrease in total mononuclear cells as a result. The difference between the retina and optic nerve response to an ONC was clear in the CX3CR1^YFP+^ microglia, which expanded in the optic nerve, but showed little difference in the retina. Despite the strong responses in GFP^lo^ and GFP^hi^ cells in the optic nerve post-full ONT, these responses are not reflected in the retina, whereas a partial transection led to a stronger response in retina. The results are consistent with the ability of the microglia in the optic nerve to proliferate, yielding both GFP^lo^ and GFP^hi^ cells consistent with the degree of injury. We did not find myeloid cells that were CD11b^+^ or CX3CR1^YFP+^ or CD11c^GFP+^ in the sheath. Instead, they were found in the parenchyma of the nerve. The sheath did not appear to be a significant conduit for macrophage migration, leaving the axons and their support tissue in the nerve as a pathway from nerve to retina.

Why the retina was less able than the optic nerve to generate more microglia is uncertain, but in preliminary results, we found that repopulation after ablation of retinal microglia required far more time than did repopulation in the optic nerve after ablation of optic nerve microglia. Those results are consistent with differences between retina and optic nerve in non-microglial local progenitors, but we do not have such evidence to date. Other studies have reported significant numbers of proliferating microglia and/or myeloid cells in retina following an ONT or retinal degeneration, often based on BrdU labeling [65, 66, 69, 70]. If so, this would provide a source that would be expected to diminish the role of microglia entering retina from the optic nerve. The number and location of BrdU labeled cells remains a question for us; we and others have encountered concerns for label spreading into other cells [7]. As a result, we have used antibodies to Ki67 to assess proliferation of myeloid cells in retina and optic nerve. Although we found many Ki67^+^ cells in the injured optic nerve, we found very few in retina post-optic nerve injury. To establish confidence in our Ki67 results, we included our control findings in retinal wholemounts post-ONC, compared to autoimmune retinitis. Ki67^+^ cells were numerous in inflamed retina, even at the very early stage of disease shown here. In unpublished results, we depleted host microglia by use of radiation bone marrow chimeras and found many Ki67^+^CD11b^+^ donor-derived macrophages in retina as it repopulated. These results suggested that our staining protocol was working but that recruited cells had much more proliferative potential. As a result, we have some confidence in our application of the Ki67 staining technique, but have not found results comparable to some other published reports, as noted above. There are many reports of proliferating microglia in brain, but our preliminary ablation results indicate that brain and retina may not be equivalent in this regard. Another potential source of retinal macrophages post-injury is recruitment from the circulation, and an intact blood-retinal barrier may reduce recruitment into and then through an injured optic nerve to the retina [13, 22]. Our parabiosis experiment showed that recruitment from the circulation was not a factor, in the case of optic nerve injury. However, the significance of this concern clearly applies to other injuries as shown from the results of Wong et al. who observed proliferation of infiltrating monocytes following RPE injury [40].

Since the optic nerve sheath did not appear to be a pathway to the retina, microglia may instead respond to chemotactic signals from astrocytes, and move along the axons, whether injured or not. Astrocytes bundle the axons as they enter the optic nerve head and influence the topography of RGC axon development and their path into the nerve [12, 55, 59]. Perhaps astrocyte processes provide scaffolding for microglia migration. Astrocytes are known to produce pro-inflammatory molecules and interact with microglia [36], and inhibition of astrocyte reactivity can have an adverse effect on function post-ONC [60]. Oligodendrocytes myelinating the mouse ON participate in the responses of microglia and astrocytes to damage in the optic nerve [8]. Their role in vectoral microglia migration through the optic nerve injury model is uncertain. In any case, a full ONT would leave no path, with the cut site as a barrier to migration. Microglia on the proximal side of the cut site could still migrate into retina and associate directly with injured axons. We found that the proximal end of a full cut optic nerve was devoid of SMI-31-staining material, astrocytes (not shown) and microglia. We suggest that the microglia cleared axon debris and moved into the retina.

## Additional files

Additional file 1: **Figure S1.** Flatmounted optic nerve 7 days post-ONC from a CD11c^GFP^ mouse. The yellow bar marks the crush site. Tissue was stained for CD11b = red; GFP = green. (DOCX 515 kb)

Additional file 2: **Figure S2.** An optic nerve transection sparing the ophthalmic artery preserved the retina and led to the appearance of a GFP^hi^ cell population in retina in flow cytometry. **a & b** Histopathology, H&E staining of mouse eyes. **a** Partial ONT that spared the ophthalmic artery. **b** 5 days post-transection of the ophthalmic artery and nerve during ONT surgery (ON&AT). **c & d** Representative flow cytometry plots of retinal cells including viable CD45^med^CD11b^hi^F4/80^+^Ly6G^−^ cells. **c** Normal control retina. **d** Transection of the optic nerve but not the artery stimulated the appearance of GFP^hi^ cells at 6 days post-ONT. (DOCX 441 kb)

Additional file 3: **Figure S3.** Although the naive NFL/RGC was sparsely populated with microglia (Manuscript Fig. 5), optical sections from slightly deeper than the NFL/RGC revealed many CX3CR1-YFP^+^ cells. Our interpretation was that we had penetrated into the IPL, consistent with the remaining small area of faint magenta staining for β3-tubulin in the upper right quadrant. Counts from the NFL/RGC and IPL revealed substantial differences in microglia numbers in naive retina (Note Manuscript Fig. 6). Yellow = CX3CR1-YFP; Magenta = β3-tubulin. (DOCX 1183 kb)

Additional file 4: **Figure S4.** Retina flat mounts from CX3CR1^YFP^:CD11c^GFP^ mice illustrate the GFP^hi^ and GFP^lo^ microglia response in the contralateral retina at days 6, 10, and 21 after an ONT in the ipsilateral retina. **a** Appearance of GFP^hi^ cells in the contralateral central retina 6 and 10 days after a full ONT. Red = β3-tubulin; Yellow = YFP; Green = GFP. 100 μm scale bars are shown on the top panels. White arrows point to the ONH. **b** Contralateral retinal flatmounts at 21 days post-partial ONT showed the progression of the GFP^hi^ cell response in the NFL/RGC at 21 d post-ONT. Note that at day 21 post-ONT the contra retina has a number of CD11b^+^ cells, but relatively few are GFP^hi^. (DOCX 2087 kb)

Additional file 5: **Figure S5.** Presence of GFP^hi^ microglia in peripheral retina of the ipsilateral and contralateral eyes at 10 days post-partial ONT. **a** Infiltration of peripheral retina with GFP^hi^ cells showed close association with affected nerve fibers. **b** Mid-peripheral retina also showed the GFP^hi^ cell association with RGC and axons whereas the contralateral retina showed fewer GFP^hi^ microglia and little close contact with the nerve fibers. Red = β3 tubulin; Green = GFP; Yellow = YFP. (DOCX 1222 kb)

Additional file 6: **Figure S6.** Parameters for counting the GFP^hi^ and GFP^lo^ microglia in the layers of the retina (see manuscript Fig. 6). Cells designated as ‘adjacent’ to the NFL are shown in part **a**, where they can be seen to be near the nerve fibers and RGC soma. A cell designated as in ‘contact’ with the NFL is shown in part **b**; it is directly associated with the nerve fiber it is on. Part **c** shows the arrangement of counting areas on a flatmounted retina, with 4 central regions and 4 peripheral regions. (DOCX 438 kb)

## References

[1] Ajami B, Bennett JL, Krieger C, Tetzlaff W, Rossi FM (2007). Local self-renewal can sustain CNS microglia maintenance and function throughout adult life. Nat Neurosci.

[2] Almasieh M, Wilson AM, Morquette B, Cueva Vargas JL, Di Polo A (2012). The molecular basis of retinal ganglion cell death in glaucoma. Prog Retin Eye Res.

[3] Apara A, Galvao J, Wang Y, Blackmore M, Trillo A, Iwao K, Brown DP Jr, Fernandes KA, Huang A, Nguyen T, Ashouri M, Zhang X, Shaw PX, Kunzevitzky NJ, Moore DL, Libby RT, Goldberg JL (2017) KLF9 and JNK3 interact to suppress axon regeneration in the adult CNS. J Neurosci. 10.1523/JNEUROSCI.0643-16.2017 10.1523/JNEUROSCI.0643-16.2017 PMC5628408 28871032

[4] Banchereau J, Steinman RM (1998). Dendritic cells and the control of immunity. Nature.

[5] Blanch RJ, Ahmed Z, Berry M, Scott RA, Logan A (2012). Animal models of retinal injury. Invest Ophthalmol Vis Sci.

[6] Bruttger J, Karram K, Wortge S, Regen T, Marini F, Hoppmann N, Klein M, Blank T, Yona S, Wolf Y, Mack M, Pinteaux E, Muller W, Zipp F, Binder H, Bopp T, Prinz M, Jung S, Waisman A (2015). Genetic cell ablation reveals clusters of local self-renewing microglia in the mammalian central nervous system. Immunity.

[7] Burns TC, Ortiz-Gonzalez XR, Gutierrez-Perez M, Keene CD, Sharda R, Demorest ZL, Jiang Y, Nelson-Holte M, Soriano M, Nakagawa Y, Luquin MR, Garcia-Verdugo JM, Prosper F, Low WC, Verfaillie CM (2006). Thymidine analogs are transferred from prelabeled donor to host cells in the central nervous system after transplantation: a word of caution. Stem Cells.

[8] Domingues HS, Portugal CC, Socodato R, Relvas JB (2016). Oligodendrocyte, astrocyte, and microglia crosstalk in myelin development, damage, and repair. Front Cell Dev Biol.

[9] Elmore MR, Najafi AR, Koike MA, Dagher NN, Spangenberg EE, Rice RA, Kitazawa M, Matusow B, Nguyen H, West BL, Green KN (2014). Colony-stimulating factor 1 receptor signaling is necessary for microglia viability, unmasking a microglia progenitor cell in the adult brain. Neuron.

[10] Fernandes KA, Harder JM, Fornarola LB, Freeman RS, Clark AF, Pang IH, John SW, Libby RT (2012). JNK2 and JNK3 are major regulators of axonal injury-induced retinal ganglion cell death. Neurobiol Dis.

[11] Fernandes KA, Harder JM, Kim J, Libby RT (2013). JUN regulates early transcriptional responses to axonal injury in retinal ganglion cells. Exp Eye Res.

[12] Fitzgibbon T, Reese BE (1996). Organization of retinal ganglion cell axons in the optic fiber layer and nerve of fetal ferrets. Vis Neurosci.

[13] Garcia-Valenzuela E, Sharma SC (1999). Laminar restriction of retinal macrophagic response to optic nerve axotomy in the rat. J Neurobiol.

[14] Ginhoux F, Greter M, Leboeuf M, Nandi S, See P, Gokhan S, Mehler MF, Conway SJ, Ng LG, Stanley ER, Samokhvalov IM, Merad M (2010). Fate mapping analysis reveals that adult microglia derive from primitive macrophages. Science.

[15] Goldmann T, Wieghofer P, Jordao MJ, Prutek F, Hagemeyer N, Frenzel K, Amann L, Staszewski O, Kierdorf K, Krueger M, Locatelli G, Hochgerner H, Zeiser R, Epelman S, Geissmann F, Priller J, Rossi FM, Bechmann I, Kerschensteiner M, Linnarsson S, Jung S, Prinz M (2016). Origin, fate and dynamics of macrophages at central nervous system interfaces. Nat Immunol.

[16] Gomez-Nicola D, Fransen NL, Suzzi S, Perry VH (2013). Regulation of microglial proliferation during chronic neurodegeneration. J Neurosci.

[17] Gregerson DS, Sam TN, McPherson SW (2004). The antigen-presenting activity of fresh, adult parenchymal microglia and perivascular cells from retina. J Immunol.

[18] Heuss ND, Lehmann U, Norbury CC, McPherson SW, Gregerson DS (2012). Local activation of dendritic cells alters the pathogenesis of autoimmune disease in the retina. J Immunol.

[19] Heuss ND, Pierson MJ, Montaniel K, McPherson SW, Lehmann U, Hussong SA, Ferrington DA, Low WC, Gregerson DS (2014). Retinal dendritic cell recruitment, but not function, was inhibited in MyD88 and TRIF deficient mice. J Neuroinflammation.

[20] Horai R, Chong WP, Zhou R, Chen J, Silver PB, Agarwal RK, Caspi RR (2015). Spontaneous ocular autoimmunity in mice expressing a transgenic T cell receptor specific to retina: a tool to dissect mechanisms of uveitis. Curr Mol Med.

[21] Horai R, Silver PB, Chen J, Agarwal RK, Chong WP, Jittayasothorn Y, Mattapallil MJ, Nguyen S, Natarajan K, Villasmil R, Wang P, Karabekian Z, Lytton SD, Chan CC, Caspi RR (2013). Breakdown of immune privilege and spontaneous autoimmunity in mice expressing a transgenic T cell receptor specific for a retinal autoantigen. J Autoimmun.

[22] Hou B, You SW, Wu MM, Kuang F, Liu HL, Jiao XY, Ju G (2004). Neuroprotective effect of inosine on axotomized retinal ganglion cells in adult rats. Invest Ophthalmol Vis Sci.

[23] Huang Y, Xu Z, Xiong S, Qin G, Sun F, Yang J, Yuan TF, Zhao L, Wang K, Liang YX, Fu L, Wu T, So KF, Rao Y, Peng B (2018). Dual extra-retinal origins of microglia in the model of retinal microglia repopulation. Cell Discov.

[24] Huang Y, Xu Z, Xiong S, Sun F, Qin G, Hu G, Wang J, Zhao L, Liang YX, Wu T, Lu Z, Humayun MS, So KF, Pan Y, Li N, Yuan TF, Rao Y, Peng B (2018). Repopulated microglia are solely derived from the proliferation of residual microglia after acute depletion. Nat Neurosci.

[25] Jeon CJ, Strettoi E, Masland RH (1998). The major cell populations of the mouse retina. J Neurosci.

[26] Joly S, Francke M, Ulbricht E, Beck S, Seeliger M, Hirrlinger P, Hirrlinger J, Lang KS, Zinkernagel M, Odermatt B, Samardzija M, Reichenbach A, Grimm C, Reme CE (2009). Cooperative phagocytes: resident microglia and bone marrow immigrants remove dead photoreceptors in retinal lesions. Am J Pathol.

[27] Jung S, Unutmaz D, Wong P, Sano G, De los Santos K, Sparwasser T, Wu S, Vuthoori S, Ko K, Zavala F, Pamer EG, Littman DR, Lang RA (2002). In vivo depletion of CD11c+ dendritic cells abrogates priming of CD8+ T cells by exogenous cell-associated antigens. Immunity.

[28] Kamran P, Sereti KI, Zhao P, Ali SR, Weissman IL, Ardehali R (2013) Parabiosis in mice: a detailed protocol. J Vis Exp. 10.3791/50556 10.3791/50556 PMC3938334 24145664

[29] Kaneko H, Nishiguchi KM, Nakamura M, Kachi S, Terasaki H (2008). Characteristics of bone marrow-derived microglia in the normal and injured retina. Invest Ophthalmol Vis Sci.

[30] Kierdorf K, Erny D, Goldmann T, Sander V, Schulz C, Perdiguero EG, Wieghofer P, Heinrich A, Riemke P, Holscher C, Muller DN, Luckow B, Brocker T, Debowski K, Fritz G, Opdenakker G, Diefenbach A, Biber K, Heikenwalder M, Geissmann F, Rosenbauer F, Prinz M (2013). Microglia emerge from erythromyeloid precursors via Pu.1- and Irf8-dependent pathways. Nat Neurosci.

[31] Kierdorf K, Katzmarski N, Haas CA, Prinz M (2013). Bone marrow cell recruitment to the brain in the absence of irradiation or parabiosis bias. PLoS One.

[32] Kokona D, Haner NU, Ebneter A, Zinkernagel MS (2017). Imaging of macrophage dynamics with optical coherence tomography in anterior ischemic optic neuropathy. Exp Eye Res.

[33] Lehmann U, Heuss ND, McPherson SW, Roehrich H, Gregerson DS (2010). Dendritic cells are early responders to retinal injury. Neurobiol Dis.

[34] Levkovitch-Verbin H, Quigley HA, Martin KR, Zack DJ, Pease ME, Valenta DF (2003). A model to study differences between primary and secondary degeneration of retinal ganglion cells in rats by partial optic nerve transection. Invest Ophthalmol Vis Sci.

[35] Li Y, Schlamp CL, Nickells RW (1999). Experimental induction of retinal ganglion cell death in adult mice. Invest Ophthalmol Vis Sci.

[36] Liddelow SA, Barres BA (2017). Reactive astrocytes: production, function, and therapeutic potential. Immunity.

[37] Lin S, Liang Y, Zhang J, Bian C, Zhou H, Guo Q, Xiong Y, Li S, Su B (2012). Microglial TIR-domain-containing adapter-inducing interferon-beta (TRIF) deficiency promotes retinal ganglion cell survival and axon regeneration via nuclear factor-kappaB. J Neuroinflammation.

[38] London A, Itskovich E, Benhar I, Kalchenko V, Mack M, Jung S, Schwartz M (2011). Neuroprotection and progenitor cell renewal in the injured adult murine retina requires healing monocyte-derived macrophages. J Exp Med.

[39] Lorber B, Tassoni A, Bull ND, Moschos MM, Martin KR (2012). Retinal ganglion cell survival and axon regeneration in WldS transgenic rats after optic nerve crush and lens injury. BMC Neurosci.

[40] Ma W, Zhang Y, Gao C, Fariss RN, Tam J, Wong WT (2017). Monocyte infiltration and proliferation reestablish myeloid cell homeostasis in the mouse retina following retinal pigment epithelial cell injury. Sci Rep.

[41] Mac Nair CE, Fernandes KA, Schlamp CL, Libby RT, Nickells RW (2014). Tumor necrosis factor alpha has an early protective effect on retinal ganglion cells after optic nerve crush. J Neuroinflammation.

[42] Mac Nair CE, Nickells RW (2015). Neuroinflammation in Glaucoma and optic nerve damage. Prog Mol Biol Transl Sci.

[43] Mac Nair CE, Schlamp CL, Montgomery AD, Shestopalov VI, Nickells RW (2016). Retinal glial responses to optic nerve crush are attenuated in Bax-deficient mice and modulated by purinergic signaling pathways. J Neuroinflammation.

[44] Madisen L, Zwingman TA, Sunkin SM, Oh SW, Zariwala HA, Gu H, Ng LL, Palmiter RD, Hawrylycz MJ, Jones AR, Lein ES, Zeng H (2010). A robust and high-throughput Cre reporting and characterization system for the whole mouse brain. Nat Neurosci.

[45] Mattapallil MJ, Wawrousek EF, Chan CC, Zhao H, Roychoudhury J, Ferguson TA, Caspi RR (2012). The Rd8 mutation of the Crb1 gene is present in vendor lines of C57BL/6N mice and embryonic stem cells, and confounds ocular induced mutant phenotypes. Invest Ophthalmol Vis Sci.

[46] McPherson SW, Heuss ND, Pierson MJ, Gregerson DS (2014). Retinal antigen-specific regulatory T cells protect against spontaneous and induced autoimmunity and require local dendritic cells. J Neuroinflammation.

[47] Merad M, Sathe P, Helft J, Miller J, Mortha A (2013). The dendritic cell lineage: ontogeny and function of dendritic cells and their subsets in the steady state and the inflamed setting. Annu Rev Immunol.

[48] Norsworthy MW, Bei F, Kawaguchi R, Wang Q, Tran NM, Li Y, Brommer B, Zhang Y, Wang C, Sanes JR, Coppola G, He Z (2017). Sox11 expression promotes regeneration of some retinal ganglion cell types but kills others. Neuron.

[49] Okabe M, Ikawa M, Kominami K, Nakanishi T, Nishimune Y (1997). 'Green mice' as a source of ubiquitous green cells. FEBS Lett.

[50] O'Koren EG, Mathew R, Saban DR (2016). Fate mapping reveals that microglia and recruited monocyte-derived macrophages are definitively distinguishable by phenotype in the retina. Sci Rep.

[51] Parkhurst CN, Yang G, Ninan I, Savas JN, Yates JR, Lafaille JJ, Hempstead BL, Littman DR, Gan WB (2013). Microglia promote learning-dependent synapse formation through brain-derived neurotrophic factor. Cell.

[52] Radius RL, Anderson DR (1979). The course of axons through the retina and optic nerve head. Arch Ophthalmol.

[53] Reyes NJ, O'Koren EG, Saban DR (2017) New insights into mononuclear phagocyte biology from the visual system. Nat Rev Immunol 17:322–332. 10.1038/nri.2017.13 10.1038/nri.2017.13 PMC5810371 28345586

[54] Rohrer B, Lohr HR, Humphries P, Redmond TM, Seeliger MW, Crouch RK (2005). Cone opsin mislocalization in RPE65−/− mice: a defect that can be corrected by 11-cis retinal. Invest Ophthalmol Vis Sci.

[55] Salinas-Navarro M, Alarcon-Martinez L, Valiente-Soriano FJ, Ortin-Martinez A, Jimenez-Lopez M, Aviles-Trigueros M, Villegas-Perez MP, de la Villa P, Vidal-Sanz M (2009). Functional and morphological effects of laser-induced ocular hypertension in retinas of adult albino Swiss mice. Mol Vis.

[56] Sandvig A, Sandvig I, Berry M, Olsen O, Pedersen TB, Brekken C, Thuen M (2011). Axonal tracing of the normal and regenerating visual pathway of mouse, rat, frog, and fish using manganese-enhanced MRI (MEMRI). J Magn Reson Imaging.

[57] Schneble N, Muller J, Kliche S, Bauer R, Wetzker R, Bohmer FD, Wang ZQ, Muller JP (2017). The protein-tyrosine phosphatase DEP-1 promotes migration and phagocytic activity of microglial cells in part through negative regulation of fyn tyrosine kinase. Glia.

[58] Steinman RM (2007). Lasker basic medical research award. Dendritic cells: versatile controllers of the immune system. Nat Med.

[59] Sun D, Lye-Barthel M, Masland RH, Jakobs TC (2009). The morphology and spatial arrangement of astrocytes in the optic nerve head of the mouse. J Comp Neurol.

[60] Sun D, Moore S, Jakobs TC (2017). Optic nerve astrocyte reactivity protects function in experimental glaucoma and other nerve injuries. J Exp Med.

[61] Tang PH, Fan J, Goletz PW, Wheless L, Crouch RK (2010). Effective and sustained delivery of hydrophobic retinoids to photoreceptors. Invest Ophthalmol Vis Sci.

[62] Tang PH, Pierson MJ, Heuss ND, Gregerson DS (2017). A subpopulation of activated retinal macrophages selectively migrated to regions of cone photoreceptor stress, but had limited effect on cone death in a mouse model for type 2 Leber congenital amaurosis. Mol Cell Neurosci.

[63] Telford WG, Hawley T, Subach F, Verkhusha V, Hawley RG (2012). Flow cytometry of fluorescent proteins. Methods.

[64] Ulland TK, Wang Y, Colonna M (2015). Regulation of microglial survival and proliferation in health and diseases. Semin Immunol.

[65] Wohl SG, Schmeer CW, Friese T, Witte OW, Isenmann S (2011). In situ dividing and phagocytosing retinal microglia express nestin, vimentin, and NG2 in vivo. PLoS One.

[66] Wohl SG, Schmeer CW, Witte OW, Isenmann S (2010). Proliferative response of microglia and macrophages in the adult mouse eye after optic nerve lesion. Invest Ophthalmol Vis Sci.

[67] Xu H, Chen M, Mayer EJ, Forrester JV, Dick AD (2007). Turnover of resident retinal microglia in the normal adult mouse. Glia.

[68] Yin Y, Henzl MT, Lorber B, Nakazawa T, Thomas TT, Jiang F, Langer R, Benowitz LI (2006). Oncomodulin is a macrophage-derived signal for axon regeneration in retinal ganglion cells. Nat Neurosci.

[69] Yuan TF, Liang YX, Peng B, Lin B, So KF (2015). Local proliferation is the main source of rod microglia after optic nerve transection. Sci Rep.

[70] Zeiss CJ, Johnson EA (2004). Proliferation of microglia, but not photoreceptors, in the outer nuclear layer of the rd-1 mouse. Invest Ophthalmol Vis Sci.

[71] Zhang Y, Zhao L, Wang X, Ma W, Lazere A, Qian HH, Zhang J, Abu-Asab M, Fariss RN, Roger JE, Wong WT (2018). Repopulating retinal microglia restore endogenous organization and function under CX3CL1-CX3CR1 regulation. Sci Adv.

