# Supplementary material for: Optic nerve as a source of activated retinal microglia post-injury
Source: Acta Neuropathol Commun. 2018 Jul 23;6:66. doi: 10.1186/s40478-018-0571-8 (PMC6055350; doi:10.1186/s40478-018-0571-8)
Supplement: Supplementary file 1 — Figure S1. Flatmounted optic nerve 7 days post-ONC from a CD11cGFP mouse. The yellow bar marks the crush site. Tissue was stained for CD11b = red; GFP = green. (DOCX 515 kb) [file 40478_2018_571_MOESM1_ESM.docx]

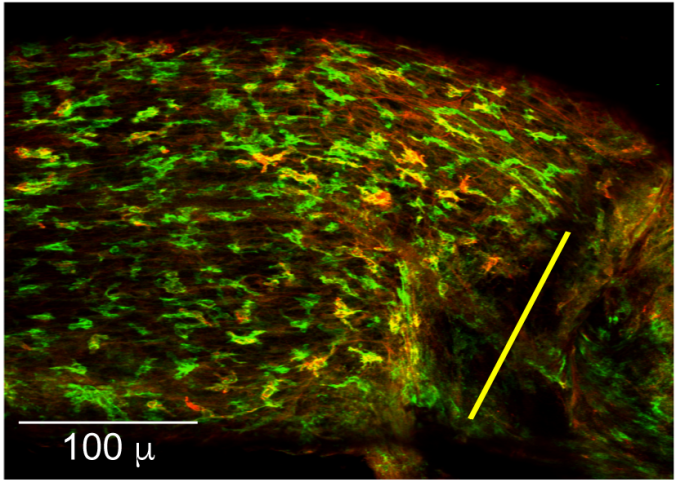


Figure S1. Additional File 1.

Flatmounted optic nerve 7 days post-ONC from a CD11c^GFP^ mouse. The yellow bar marks the crush site. Tissue was stained for CD11b = red; GFP = green.
