# Supplementary material for: Optic nerve as a source of activated retinal microglia post-injury
Source: Acta Neuropathol Commun. 2018 Jul 23;6:66. doi: 10.1186/s40478-018-0571-8 (PMC6055350; doi:10.1186/s40478-018-0571-8)
Supplement: Supplementary file 2 — Figure S2. An optic nerve transection sparing the ophthalmic artery preserved the retina and led to the appearance of a GFPhi cell population in retina in flow cytometry. a & b Histopathology, H&E staining of mouse eyes. a Partial ONT that spared the ophthalmic artery. b 5 days post-transection of the ophthalmic artery and nerve during ONT surgery (ON&AT). c & d Representative flow cytometry plots of retinal cells including viable CD45medCD11bhiF4/80+Ly6G− cells. c Normal control retina. d Transection of the optic nerve but not the artery stimulated the appearance of GFPhi cells at 6 days post-ONT. (DOCX 441 kb) [file 40478_2018_571_MOESM2_ESM.docx]

**
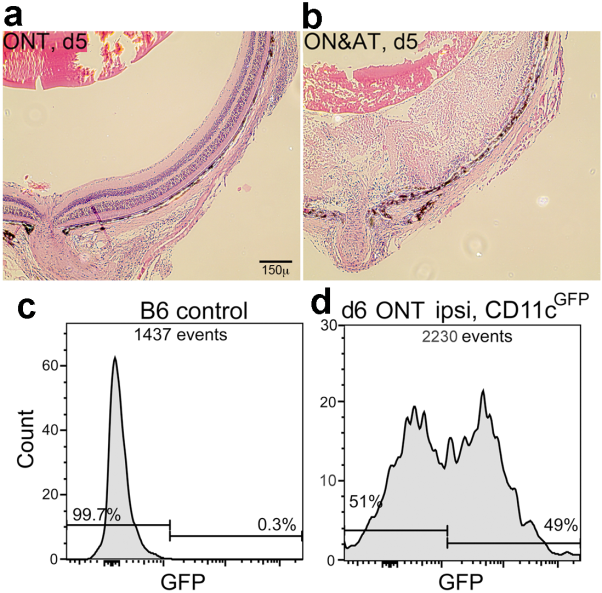
**

Figure S2. Additional File 2.

An optic nerve transection sparing the ophthalmic artery preserved the retina and led to the appearance of a GFP^hi^ cell population in retina in flow cytometry. **a & b** Histopathology, H&E staining of mouse eyes. **a** Partial ONT that spared the ophthalmic artery. **b** Five days post-transection of the ophthalmic artery and nerve during ONT surgery (ON&AT). **c & d** Representative flow cytometry plots of retinal cells including viable CD45^med^CD11b^hi^F4/80^+^Ly6G^-^ cells. **c** Normal control retina. **d** Transection of the optic nerve but not the artery stimulated the appearance of GFP^hi^ cells at 6 days post-ONT.
