# Supplementary material for: Optic nerve as a source of activated retinal microglia post-injury
Source: Acta Neuropathol Commun. 2018 Jul 23;6:66. doi: 10.1186/s40478-018-0571-8 (PMC6055350; doi:10.1186/s40478-018-0571-8)
Supplement: Supplementary file 3 — Figure S3. Although the naive NFL/RGC was sparsely populated with microglia (Manuscript Fig. 5), optical sections from slightly deeper than the NFL/RGC revealed many CX3CR1-YFP+ cells. Our interpretation was that we had penetrated into the IPL, consistent with the remaining small area of faint magenta staining for β3-tubulin in the upper right quadrant. Counts from the NFL/RGC and IPL revealed substantial differences in microglia numbers in naive retina (Note Manuscript Fig. 6). Yellow = CX3CR1-YFP; Magenta = β3-tubulin. (DOCX 1183 kb) [file 40478_2018_571_MOESM3_ESM.docx]

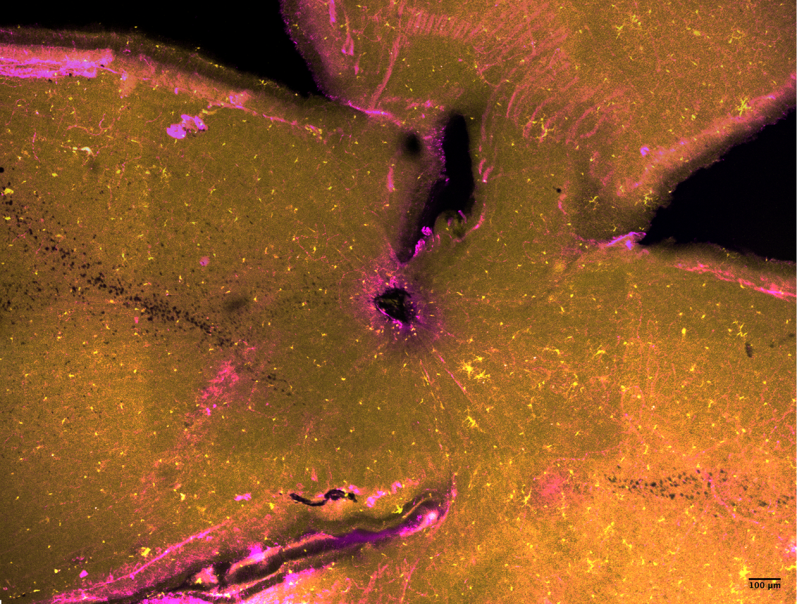


Figure S3. Additional File 3.

Although the naive NFL/RGC was sparsely populated with microglia (Manuscript Fig. 5), optical sections from slightly deeper than the NFL/RGC revealed many CX3CR1-YFP^+^ cells. Our interpretation was that we had penetrated into the IPL, consistent with the remaining small area of faint magenta staining for β3-tubulin in the upper right quadrant. Counts from the NFL/RGC and IPL revealed substantial differences in microglia numbers in naive retina (Note Manuscript Fig. 6). Yellow = CX3CR1-YFP; Magenta = β3-tubulin
