# Supplementary material for: Optic nerve as a source of activated retinal microglia post-injury
Source: Acta Neuropathol Commun. 2018 Jul 23;6:66. doi: 10.1186/s40478-018-0571-8 (PMC6055350; doi:10.1186/s40478-018-0571-8)
Supplement: Supplementary file 4 — Figure S4. Retina flat mounts from CX3CR1YFP:CD11cGFP mice illustrate the GFPhi and GFPlo microglia response in the contralateral retina at days 6, 10, and 21 after an ONT in the ipsilateral retina. a Appearance of GFPhi cells in the contralateral central retina 6 and 10 days after a full ONT. Red = β3-tubulin; Yellow = YFP; Green = GFP. 100 μm scale bars are shown on the top panels. White arrows point to the ONH. b Contralateral retinal flatmounts at 21 days post-partial ONT showed the progression of the GFPhi cell response in the NFL/RGC at 21 d post-ONT. Note that at day 21 post-ONT the contra retina has a number of CD11b+ cells, but relatively few are GFPhi. (DOCX 2087 kb) [file 40478_2018_571_MOESM4_ESM.docx]

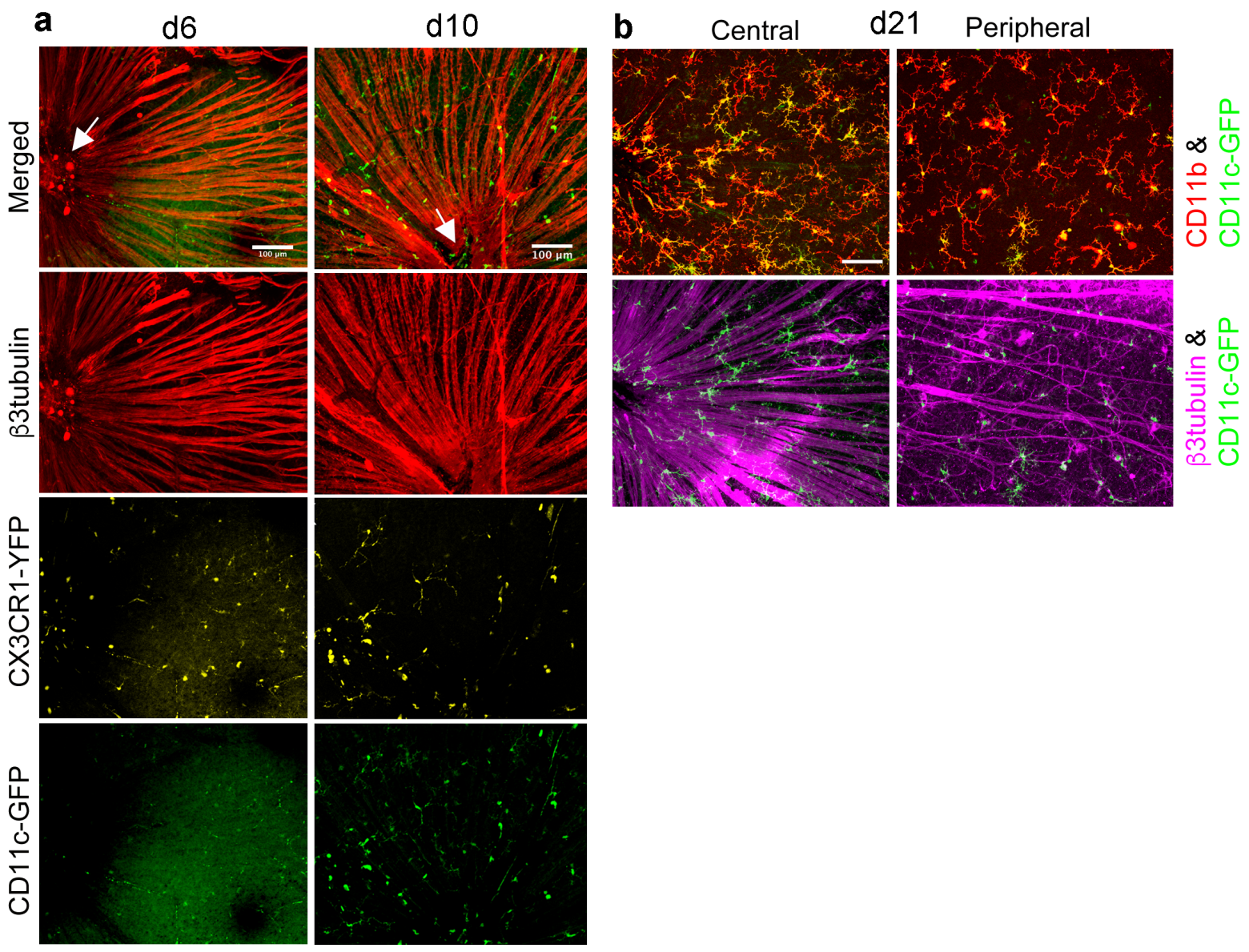
Figure S4. Additional File 4.

Retina flat mounts from CX3CR1^YFP^:CD11c^GFP^ mice illustrate the GFP^hi^ and GFP^lo^ microglia response in the contralateral retina at days 6, 10, and 21 after an ONT in the ipsilateral retina. **a** Appearance of GFP^hi^ cells in the contralateral central retina 6 and 10 days after a full ONT. Red = β3-tubulin; Yellow = YFP; Green = GFP. 100 micron scale bars are shown on the top panels. White arrows point to the ONH. **b** Contralateral retinal flatmounts at 21 days post-partial ONT showed the progression of the GFP^hi^ cell response in the NFL/RGC at 21 d post-ONT. Note that at day 21 post-ONT the contra retina has a number of CD11b^+^ cells, but relatively few are GFP^hi^.
