# Supplementary material for: Optic nerve as a source of activated retinal microglia post-injury
Source: Acta Neuropathol Commun. 2018 Jul 23;6:66. doi: 10.1186/s40478-018-0571-8 (PMC6055350; doi:10.1186/s40478-018-0571-8)
Supplement: Supplementary file 5 — Figure S5. Presence of GFPhi microglia in peripheral retina of the ipsilateral and contralateral eyes at 10 days post-partial ONT. a Infiltration of peripheral retina with GFPhi cells showed close association with affected nerve fibers. b Mid-peripheral retina also showed the GFPhi cell association with RGC and axons whereas the contralateral retina showed fewer GFPhi microglia and little close contact with the nerve fibers. Red = β3 tubulin; Green = GFP; Yellow = YFP. (DOCX 1222 kb) [file 40478_2018_571_MOESM5_ESM.docx]

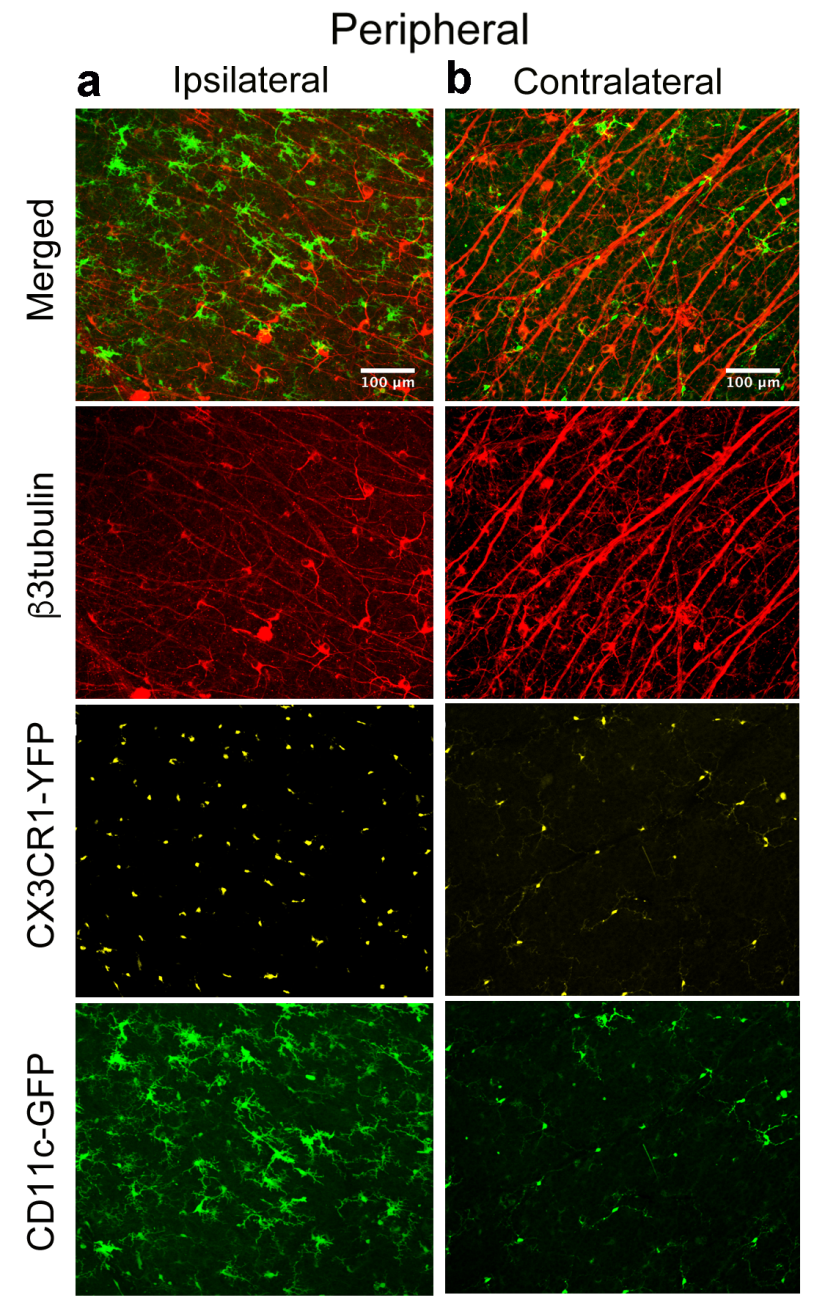


Figure S5. Additional File 5.

Presence of GFP^hi^ microglia in peripheral retina of the ipsilateral and contralateral eyes at 10 days post-partial ONT. **a** Infiltration of peripheral retina with GFP^hi^ cells showed close association with affected nerve fibers. **b** Mid-peripheral retina also showed the GFP^hi^ cell association with RGC and axons whereas the contralateral retina showed fewer GFP^hi^ microglia and little close contact with the nerve fibers. Red = β3 tubulin; Green = GFP; Yellow = YFP.
