# Supplementary material for: Optic nerve as a source of activated retinal microglia post-injury
Source: Acta Neuropathol Commun. 2018 Jul 23;6:66. doi: 10.1186/s40478-018-0571-8 (PMC6055350; doi:10.1186/s40478-018-0571-8)
Supplement: Supplementary file 6 — Figure S6. Parameters for counting the GFPhi and GFPlo microglia in the layers of the retina (see manuscript Fig. 6). Cells designated as ‘adjacent’ to the NFL are shown in part a, where they can be seen to be near the nerve fibers and RGC soma. A cell designated as in ‘contact’ with the NFL is shown in part b; it is directly associated with the nerve fiber it is on. Part c shows the arrangement of counting areas on a flatmounted retina, with 4 central regions and 4 peripheral regions. (DOCX 438 kb) [file 40478_2018_571_MOESM6_ESM.docx]

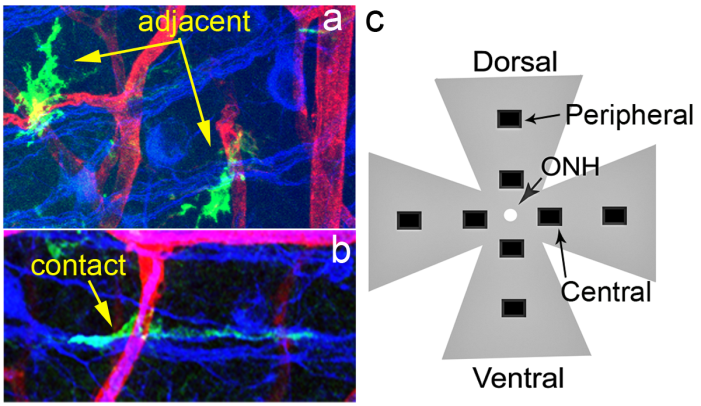


Figure S6. Additional File 6.

Parameters for counting the GFP^hi^ and GFP^lo^ microglia in the layers of the retina (see manuscript Fig. 6). Cells designated as 'adjacent' to the NFL are shown in part **a**, where they can be seen to be near the nerve fibers and RGC soma. A cell designated as in 'contact' with the NFL is shown in part **b**; it is directly associated with the nerve fiber it is on. Part **c** shows the arrangement of counting areas on a flatmounted retina, with 4 central regions and 4 peripheral regions.
